# High iASPP *(PPP1R13L)* expression is an independent predictor of adverse clinical outcome in acute myeloid leukemia (AML)

**DOI:** 10.1038/s41419-024-07190-8

**Published:** 2024-11-30

**Authors:** Mihada Bajrami Saipi, Alessia Ruiba, Marcus Matthias Schittenhelm, Gunnar Blumenstock, Balázs Győrffy, Serena Fazio, Marlon Hafner, Anna-Lena Ahrens, Lara Aldinger, Vanessa Aellig, François G. Kavelaars, César Nombela-Arrieta, Falko Fend, Peter J. M. Valk, Driessen Christoph, Kerstin Maria Kampa-Schittenhelm

**Affiliations:** 1 Department of Hematology, Oncology, Clinical Immunology and Rheumatology, University Hospital Tübingen (UKT), Tübingen, Germany; 2 Department of Medical Oncology, University Hospital Tübingen (UKT), Tübingen, Germany; 3 Medical Research Center (MFZ) and Clinic for Medical Oncology and Hematology, Cantonal Hospital St. Gallen (KSSG), St. Gallen, Switzerland; 4 on behalf of the Swiss Group for Clinical Cancer Research (SAKK), St Gallen, Switzerland; 5 Institute of Clinical Epidemiology and Applied Biometry, Eberhard Karls University Tübingen, Tübingen, Germany; 6 Semmelweis University, Department of Bioinformatics, Budapest, Hungary; 7 TTK Cancer Biomarker Research Group, Institute of Molecular Life Sciences, Budapest, Hungary; 8 Dept. of Biophysics, Medical School, University of Pecs, Pecs, Hungary; 9 Department of Medical Oncology and Hematology, University Hospital and University of Zürich, Zürich, Switzerland; 10 Department of Hematology, Erasmus MC Cancer Institute, University Medical Center Rotterdam, Rotterdam, the Netherlands; 11 on behalf of the Dutch-Belgian Hemato-Oncology Cooperative Group (HOVON), Rotterdam, the Netherlands; 12 Institute of Pathology and Neuropathology and Comprehensive Cancer Center, University Hospital Tübingen, Tübingen, Germany

**Keywords:** Acute myeloid leukaemia, Predictive markers, Apoptosis

## Abstract

Apoptosis-stimulating proteins of p53 (ASPPs) are a family of proteins that modulate key tumor suppressor pathways via direct interaction with p53. Deregulation of these proteins promotes cancer development and impairs sensitivity to systemic (chemo)therapy and radiation. In this study, we describe that the inhibitor of ASPP (iASPP) is frequently highly expressed in acute myeloid leukemia (AML) and that overexpression correlates with a poor clinical outcome. Four independent patient cohorts comprising about 1500 patient samples were analysed and consistently confirm an association of high iASPP expression with unfavourable clinical characteristics and shorter survival. Notably, the predictive role of iASPP is independent of, and adds information to, the European LeukemiaNET (ELN) risk classification. *iASPP*-interference cell models were developed to investigate the underlying functional aspects of iASPP in AML biology. Attenuation of iASPP expression resulted in reduced proliferation rates of leukemic blasts and rendered cells more susceptible towards induction of apoptosis in response to cytotoxic therapy. In line, independent NSG xenograft mouse experiments demonstrate that attenuation of iASPP results in a significant delay of disease onset and tumor burden and this translates to longer overall survival of mice. In conclusion, deregulation of iASPP has direct functional consequences in AML. Determination of iASPP expression levels provides valuable additional information as a predictive marker in AML and may guide treatment decisions.

## Introduction

Despite remarkable progress in the last decades, the majority of patients diagnosed with acute myeloid leukemia (AML) face a poor prognosis (seer.cancer.gov). Prognostic scores have been established to guide therapy for patients that are intensively treated in a curative intention (e.g., European LeukemiaNet [1] or NCCN (NCCN.org, V3.2023) [2]). Nevertheless, rates of refractory or relapsing disease remain high and even in the so called favorable risk situation patients face a considerable risk of relapse [1]. Therefore, additional or better markers are needed to predict therapy outcome and guide treatment decisions.

The p53 signalling pathway is known to be a central player in the cellular stress response and tumor control. Depending on the origin and type of stress, activation of p53 can lead to cell cycle arrest, DNA repair or senescence, autophagy and apoptosis [3]. Loss of p53 wild-type function is frequently observed in cancer. It is one of the most frequently mutated genes, with more than 60% of malignancies harboring inactivating *TP53* mutations (reviewed by Vousden and Prives [4] and UMD TP53 Mutation Database at p53.fr [5]). In AML, *TP53* mutations or chromosomal aberrations affecting the short arm of chromosome 17 are predominantly found in MDS- or therapy-related acute myeloid leukemia [6, 7] and predict for a higher risk of therapy failure and an adverse survival outcome [1, 8]. However, most de novo AML have a *TP53* wt background, and other factors apply that unfavourably influence cell integrity and apoptosis signaling.

ASPPs (ankyrin repeat domain, SH3 domain and proline-rich sequence containing proteins) are a family of p53-binding proteins, comprising two pro-apoptotic members, ASPP1 and ASPP2, and an anti-apoptotic protein, inhibitory (i)ASPP. While we have previously demonstrated that both pro-apoptotic ASPP family members are frequently deregulated in AML, and that attenuated levels of either ASPP1 or ASPP2 correlats with an adverse outcome [9, 10], the significance of iASPP in AML is unclear.

iASPP is encoded by the protein phosphatase 1 regulatory subunit 13 like gene (*PPP1R13L*, hereafter referred to as *iASPP*) [11, 12] and is an evolutionarily conserved inhibitor of p53-mediated apoptosis with independent oncoprotein functions [13]. In several cancer models, iASPP has been shown to have pro-proliferative and anti-apoptotic properties. Consequently, high levels of iASPP are associated with poor prognosis in gynaecological cancers, melanoma and glioma [14–17].

The role of iASPP in leukemia is less studied. In a transgenic mouse model, iASPP has been shown to promote long-term reconstitution and self-renewal of haematopoietic stem cells (HSCs). In line, the same study also demonstrated that iASPP prevents the induction of apoptosis in HSCs upon cellular stressors such as irradiation [18]. Physiologically, iASPP serves as a checkpoint to maintain cellular integrity, but at the same time high levels of iASPP may facilitate the accumulation of DNA damage, thus promoting oncogenesis.

In line with these findings, it has been suggested that iASPP is especially expressed in CD34^+^ leukemia blasts [19].

In this work, we aimed to elucidate iASPP expression patterns in defined AML patient cohorts and investigate the functional consequences thereof. Prognostic value of iASSP and consistency with current AML risk models were determined and validated in independent patient sets. An iASPP-interference model was established to study the direct influence of iASPP on AML biology and response to therapy in vitro as well as in a xenotransplant mouse model.

## Results

### iASPP is highly expressed in AML

We assessed *iASPP* mRNA levels in a large pan-cancer RNAseq data platform, which includes 56,938 unique samples from three well-defined datasets (TCGA/TARGET/GTEx) [20]. Focussing on the AML cohorts within this extensive dataset, the TCGA and TARGET repositories contain a total of 151 and 145 adult and pediatric patient samples.

**Fig. 1:**
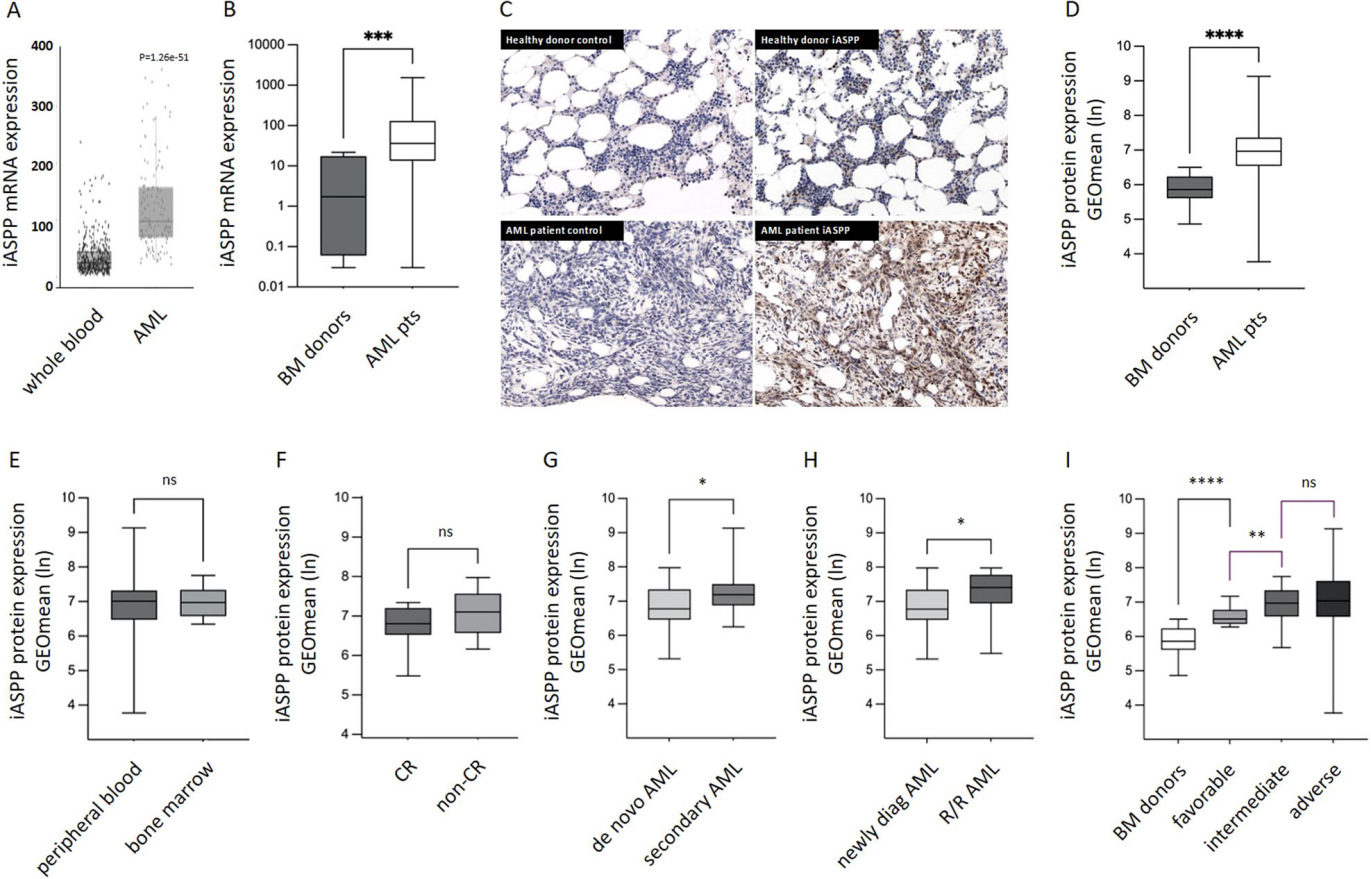
iASPP expression in AML. **A** Relative *iASPP* mRNA expression levels of a large RNAseq dataset including *n* = 296 pts with AML compared to physiologic whole blood samples, *n* = 670. **B** Relative *iASPP* mRNA expression levels of 43 AML patients compared to samples from healthy bone marrow (BM) donors (*n* = 11). *GAPDH* served as a housekeeping gene. **C** Immunohistochemistry stains (40x) of iASPP in bone marrow of a healthy donor and a patient with AML. **D** Relative iASPP protein expression levels in 73 patients with newly diagnosed AML compared to mononuclear cells from 31 healthy bone marrow (BM) donors as detected by immunostaining in a flow cytometer approach. Isotype IgG controls served as basal levels. **E** iASPP expression of leukemia blasts derived from bone marrow (*n* = 26) or peripheral blood (*n* = 55). **F**−**I** iASPP expression according to (**F**) complete remission (CR), *n* = 10, vs. pts. failing CR, *n* = 22, *p* = 0.272, (**G**) AML qualifiers (de novo AML, *n* = 42, vs. AML progressing from MDS or MDS/MPN and therapy related AML according to the ICC 2022 classification [23] (ref. to as secondary AML), *n* = 24, (**H**) newly diagnosed AML vs. R/R AML (*n* = 10) and (**I**) ELN 2017 risk stratification (healthy bm donors (*n* = 31), favorable risk (*n* = 15), intermediate risk (*n* = 19), adverse risk (*n* = 23)). ns (not significant), *p ≤ 0.05, **p ≤ 0.01, ***p ≤ 0.001, ****p ≤ 0.0001 (Mann-Whitney-U-Test).

Together, in this analysis distinct and frequent elevation of *iASPP* levels were observed when compared to physiologic whole blood samples (*n* = 670) from the GTEx dataset. Notably, individual expression levels varied widely (Fig. 1A).

Increased expression levels of *iASPP* mRNA in AML were next validated in an independent random cohort of 43 unselected patients with newly diagnosed AML, which was compared to freshly isolated mononuclear cells of a healthy bone marrow donor cohort (*n* = 11) serving as a control. Again, we found median *iASPP* mRNA expression levels to be frequently and significantly elevated in AML patients – while individual expression levels varied widely (Fig. 1B).

To determine whether the detection of *iASPP* mRNA leads to protein expression, bone marrow biopsies were immunostained for iASPP, which revealed genuine translation and expression of iASPP protein in the leukemia samples (Fig. 1C).

Next, a flow cytometry-based intracellular immunostaining approach was set up [10] to screen 73 newly diagnosed AML samples and mononuclear cells from the bone marrow of 31 healthy donors for iASPP protein expression. Consistent with the mRNA and immunohistochemistry results, a significant increase in mean iASPP protein expression levels was detected in the leukemia cohort compared to healthy donors (Fig. 1D) – whereas there was no difference in iASPP expression levels of leukemic blasts from bone marrow aspirates when compared to leukemic blasts isolated from peripheral blood (Fig. 1E). The clinical characteristics of the random patient cohort are shown in Supplementary Table S1.

To evaluate the predictive value of iASPP for therapy response in patients undergoing intensive chemotherapy, 10 patients with confirmed complete remission (CR) after the first cycle of induction therapy were analyzed in comparison to 22 patients failing CR (comprising cases with incomplete hematological recovery (CRi) after induction chemotherapy as well as patients with partial remission and refractory disease). A trend to higher iASPP expression levels was observed in the non-responding cohort – whereas significance was not reached (Fig. 1F).

In addition, all patients were classified according to AML qualifiers (de novo AML vs. AML progressing from MDS or MDS/MPN and therapy-related AML according to the ICC 2022 classification [21]) and iASPP expression levels were assessed. Statistical analysis showed formal significance with higher mean iASPP expression levels in secondary AML compared to de novo AML (*p* = 0.0149), (Fig. 1G).

Next, iASPP levels were determined in patients with newly diagnosed AML (*n* = 42) and patients with relapsed or refractory (R/R) AML (*n* = 10) - with significantly higher mean iASPP levels detected in patients with R/R AML than in the cohort of patients with de novo AML (*p* = 0.0450), (Fig. 1H).

It should be noted that statistical significance for increased mean iASPP levels was achieved in these analyses, but individual expression levels varied widely.

In an attempt to determine whether iASPP expression levels are associated with genetic risk profiles, all patients with newly diagnosed AML were classified into three risk groups according to the ELN 2017 predictive risk stratification score (which was the current risk score at the time the patients were treated) [8].

Protein levels of iASPP in AML were studied in comparison to mononuclear cells (MCs), isolated from healthy bm donors. Patients with a favorable risk profile formally showed significantly lower mean iASPP expression levels when compared to the intermediate and adverse risk groups but were still significantly higher when compared to the healthy MC controls. However, individual expression levels again varied widely in the intermediate and adverse categories, suggesting that iASPP expression patterns are not reflecting the ELN 2017 risk groups (Fig. 1I).

### iASPP expression levels correlate with clinical outcome

To further investigate whether *iASPP* expression correlates with clinical outcome, we identified a transcriptome dataset with clinical follow-up data from a large cohort of patients undergoing intensive chemotherapy (Gene Expression Omnibus (GEO) Accession GSE6891 [22]). *iASPP* expression levels were assessed in relation to survival data.

The dataset includes altogether 536 patient samples [23], average age 42.2 ± 12.1 years, 50% male (*n* = 230, of 460 with known gender), and 49 months average follow-up with 66.3% of the patients having an event (*n* = 345). Promyelocytic leukemia cases were excluded from the survival analysis.

**Fig. 2:**
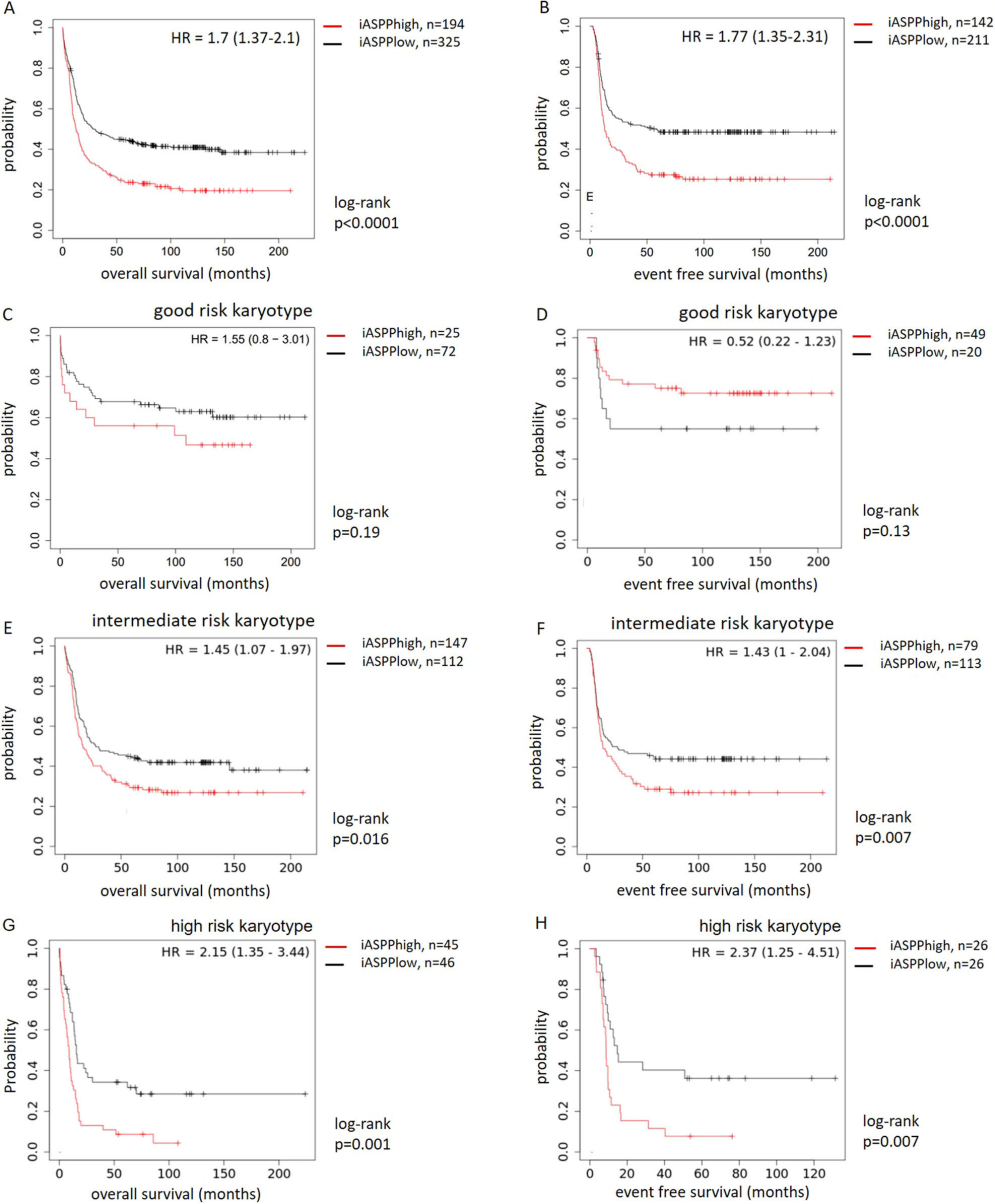
*iASPP* and survival in AML. **A**, **C**, **E**, **G** OS and **B**, **D**, **F**, **H** EFS according to *iASPP* expression and risk category according to karyotype in a transcriptomic dataset (GSE6891).

Indeed, high *iASPP* expression in this patient population correlated with an adverse overall survival (OS) and event-free survival (EFS) with hazard ratios of 1.7 (OS) and 1.77 (EFS), respectively (Fig. 2A, B). This corresponds to a median OS of 4 and 20.5 months and a median EFS of 12.9 and 54.3 months for the *iASPP*_*high*_ vs. *iASPP*_*low*_ cohorts.

Interestingly, categorising this patient population into genetic risk groups by karyotype revealed that determining *iASPP* expression level provides additional prognostic information - refining survival probabilities within the risk cohorts. A significant survival benefit was observed in the intermediate and unfavorable risk groups for patients with an *iASPP*_*low*_ expression profile when compared to *iASPP*_high_ expressors (OS_int risk_ 27.5 and 16.2 months, EFS_int risk_ 27.5 and 14.5 months and OS_high risk_ 15.7 and 9.1 months, EFS_high risk_ 14.7 and 8.7 months) (Fig. 2C–H).

In addition, risk factors by gene mutation (*NPM1*/*FLT3* ITD) were assessed – with a pattern confirming high iASPP expression as an adverse survival factor (Supplementary Fig. S1).

### iASPP expression and clinical outcome in an independent clinical validation cohort

To further confirm the predictive role of *iASPP* in AML, an additional well-defined patient cohort was assessed: samples of 274 patients treated in the HOVON102 trial [24] (EudraCT 2009-011613-24) were available for comparative analysis of *iASPP* expression patterns and clinical outcome. mRNA expression levels were assessed in relation to *GAPDH* as a housekeeping gene.

Receiver operating characteristic (ROC) analysis determined a ΔCP cut-off threshold in this cohort, which best discriminates clinical parameters according to *iASPP* expression levels with optimal diagnostic accuracy and a sensitivity of 80%.

**Fig. 3:**
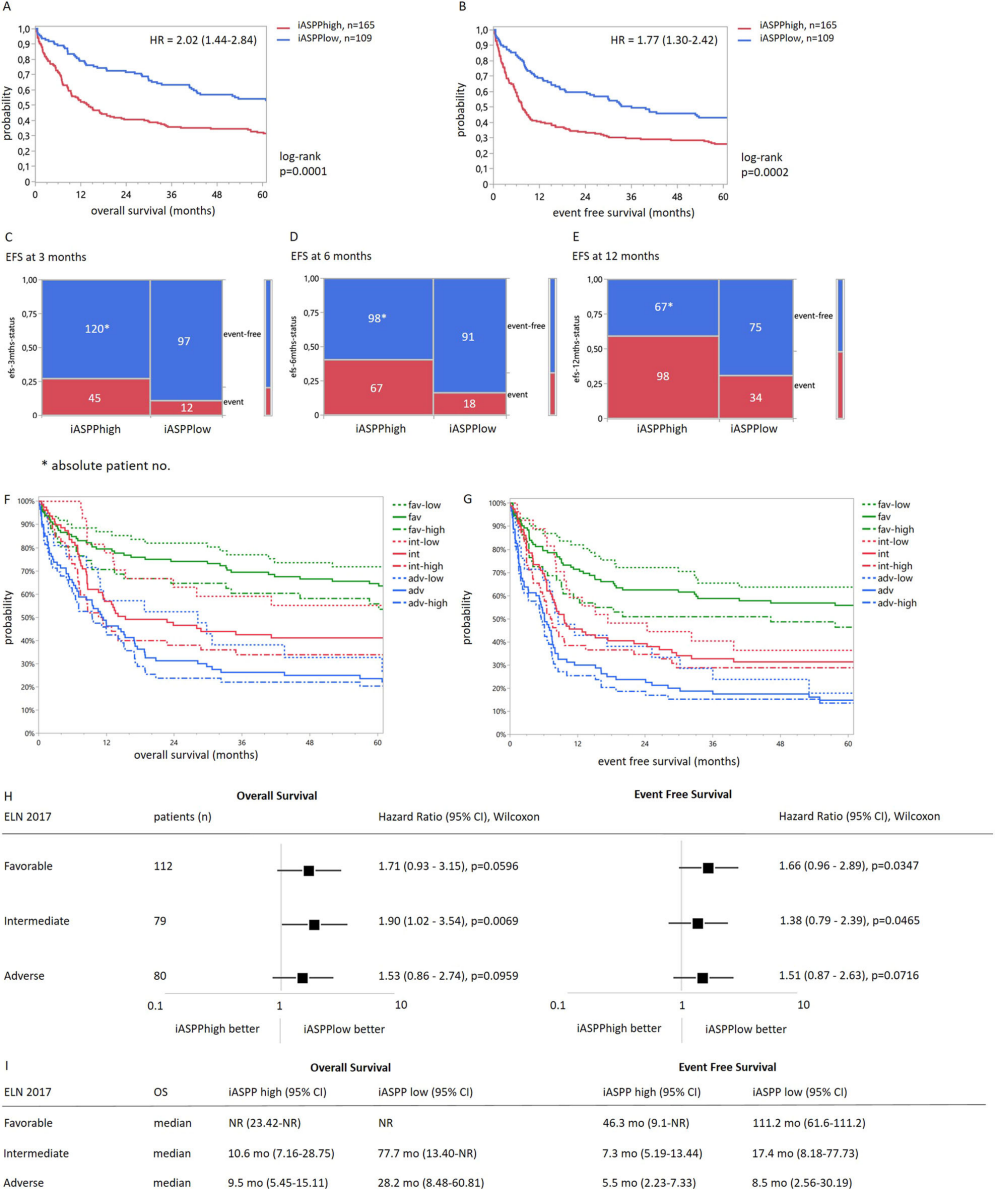
*iASPP* and survival in a patient cohort treated in the HOVON102 trial (*n* = 274). **A** OS and **B** EFS according to *iASPP* expression level. **C–E** Contingency analysis: proportion of event-free survival at 3 (**C**), 6 (**D**) and 12 (**E**) months after achieving remission. **F** OS, **G** EFS hazard ratios and **I** median OS data according to *iASPP* expression levels and ELN 2017 risk score (favorable/intermediate/adverse risk +/- *iASPP*_*low*_ or *iASPP*_*high*_).

High *iASPP* scores (i.e. with a CP cut-off < 18.25) defined a cohort of patients with an unfavorable clinical outcome compared to a cohort of patients who did not express high *iASPP* levels (median OS 14.1 mo vs. 62.1 mo, log-rank *p* = 0.0037, resp. median EFS 7.6 mo vs. 36.0 mo, log-rank *p* = 0.0106) (Fig. 3A, B), which points to a predictive role of *iASPP* in patients with newly diagnosed AML that are intensively treated.

Analysis of EFS, as the primary endpoint of the trial, demonstrated that events (i.e., therapy failure, death or relapse) in the *iASPP*_high_ cohort occur early within the first year of follow-up (Fig. 3C–E). This finding argues for a functional resistance mechanism of *iASPP* in response to chemotherapy and this aspect is modeled in the following chapter.

To further evaluate whether *iASPP* expression levels add additional information to the ELN risk classification, patients were categorized into three risk cohorts according to the ELN 2017 (consistent with Fig. 1I) and the current ELN 2022 risk classification. In particular, employing *iASPP* expression, it was possible to further refine the clinical risk profiles for OS as well as EFS in all three risk groups according to ELN 2017 (Fig. 3F, G) and ELN 2022 (Supplementary Fig. S2A, B).

Employing Cox regression analysis for ELN2017 or ELN 2022 risk groups, iASPP expression, sex and age thereby reveals that *iASPP* is an independent risk factor to predict overall survival (OS HR 0.63, 0.44–0.89, *p* < 0.0095, EFS HR 0.71, 0.52–0.98, *p* < 0.0391).

These results indicate a higher chance of survival for patients with low iASPP expression, which is independent of the ELN risk group (high statistical significance in low and intermediate risk, near significance in high risk cohorts with regard to OS and EFS, compare Fig. 3H (median OS and EFS rates are provided in Fig. 3I; 1- and 2-year OS rates are listed in Table 1). Similar survival patterns were observed when patients were categorised according to the ELN 2022 classification (Supplementary Figs.2C, D). Together, these findings provide evidence that the assessment of *iASPP* expression adds additional information to clinical outcome parameters, which is not reflected by the ELN 2017/2022 risk classification.

**Table 1:** 1- and 2-year survival rates according to ELN 2017 risk classification and iASPP expression levels.

		1- and 2- year Overall Survival	
ELN 2017		iASPP high (95% CI)	iASPP low (95% CI)
Favorable	1-year	70.6%	86.9%
	2-year	64.7%	82.0%
Intermediate	1-year	45.9%	77.8%
	2-year	37.9%	63.0%
Adverse	1-year	42.4%	57.1%
	2-year	23.4%	52.4%

Additional subgroup analyses were performed on selected cohorts defined by gene mutation. Here, a general negative impact of high *iASPP* expression levels on survival and early treatment failure is confirmed. However, the patient numbers were small and the predictive role of *iASPP* will need to be addressed in larger defined cohorts (Supplementary Fig. S3).

### iASPP expression correlates with leukemia biology and response to therapy in AML cell line models

To validate a biological role of deregulated iASPP in leukemogenesis and therapy resistance, two independent *iASPP* knock-down (KD) cell models were generated. First, a stable *iASPP* shRNA knock-down was established in the *TP53* wt, mutant *FLT3*-internal tandem duplication (ITD) AML cell line MOLM14, which mimics *FLT3*-mutated AML (in which high levels of *iASPP* predict for poor survival, see Supplementary Fig. S1).

**Fig. 4:**
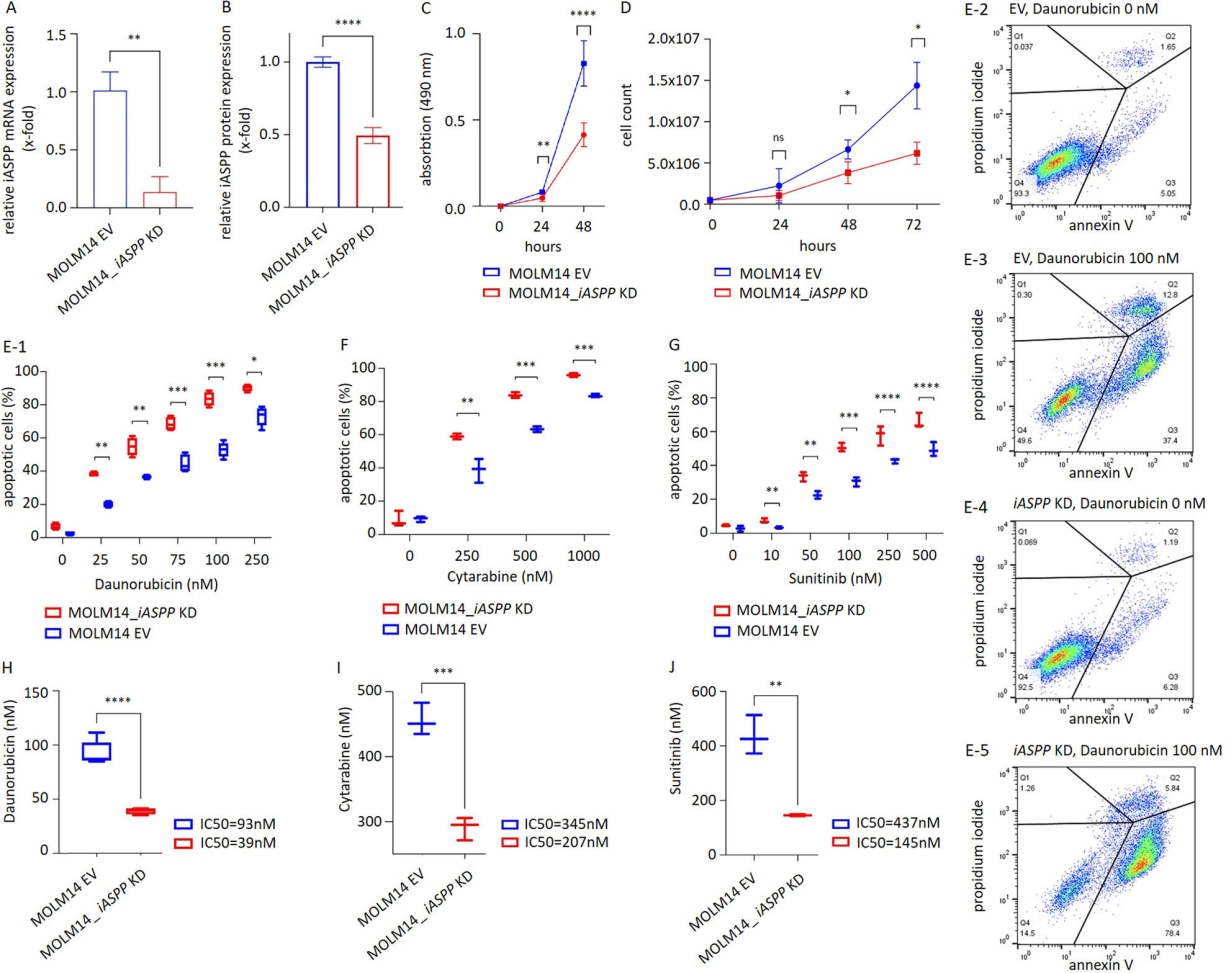
Functional analysis of iASPP in a MOLM14 interference cell line model. **A**, **B** Transduction efficiency assessed by (**A**) mRNA assessed by RT-qPCR and (**B**) protein levels assessed by flow cytometry. **C** *iASPP*-interference results in reduced metabolic activity as assessed by XTT, **D** corresponding to an attenuated proliferation rate with a prolonged cellular doubling time (16 h for *iASPP* KD vs. 13 h for *iASPP* EV). **E**, **F**, **G** Induction of apoptosis in response to daunorubicin, cytarabine or FLT3/multikinase-inhibitor sunitinib. **H**, **I**, **J** Box plot analysis to determine IC_50_ concentrations compared to empty vector (EV) control strains. Technical triplicates minimum for all assays. *p ≤ 0.05, **p ≤ 0.01, ≤ ***p ≤ 0.001, ****p ≤ 0.0001 (t-test).

Evidence of transduction efficiency with stable attenuation of iASPP mRNA and protein expression levels is shown in Fig. 4A, B.

After successful *iASPP*-interference, MOLM14_*iASPP* KD cell strains were monitored for cellular proliferation capacities. XTT viability assays were performed and data was confirmed by assessing cell doubling times compared to the EV controls.

*iASPP* KD strains showed a significant decrease in proliferation rates as reflected by reduced metabolic activity and longer cell doubling times of approximately 16 h for *iASPP* KD vs. 13 hours for the EV control strain (Fig. 4C, D). It is remarkable that a relatively modest knockdown results in significant changes in proliferation rates – strengthening the functional role of iASPP in acute myeloid leukemia.

Next, the influence of iASPP expression on apoptosis induction upon cytotoxic therapy was assessed in this MOLM14_*iASPP* KD model. Dose-dilution assays were performed using daunorubicin and cytarabine as hallmark chemotherapeutics in AML. As MOLM14 cells harbor a *FLT3* ITD mutation, which confers high sensitivity towards FLT3-inhibitors such as sunitinib [25], a targeted-therapy approach was evaluated as well. Induction of apoptosis was assessed after 48 h using an Annexin V-based flow cytometry apoptosis assay.

As expected, *iASPP*-interference rendered cells more susceptible to chemotherapy as well as FLT3-targeted therapy (Fig. 4E–G) – with a significant computed dose reduction of IC_50s_ for all tested agents (Fig. 4H–J).

We further confirmed the cytoprotective capacity of iASPP in freshly harvested native leukemia blasts of a patient with newly diagnosed AML. Indeed, *iASPP*-interference (Supplementary Fig. 4A) led to sensitization of blasts towards anthracycline (daunorubicin) treatment at a dose of 100 nM for 48 h ex vivo (Supplementary Fig. 4B).

**Fig. 5:**
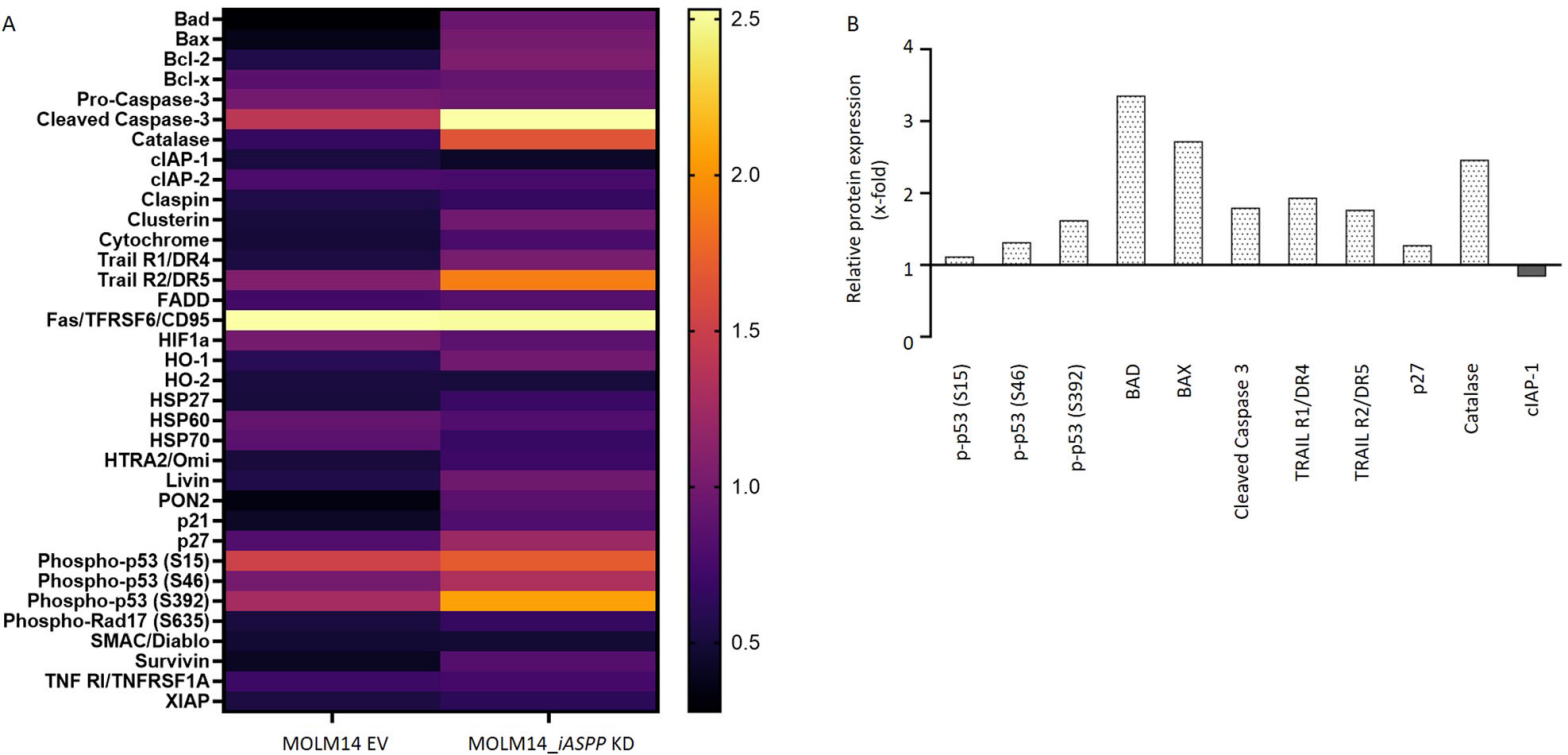
Apoptosis signaling of MOLM14 *iASPP*-interferenced vs. EV cell strains in response to daunorubicin. **A** Heat map of a proteome profiler human apoptosis array covering 35 proteins involved in apoptosis control. **B** Upregulation of proteins involved in induction of apoptosis and cell cycle control in the *iASPP* knock down (KD) vs EV cell strains.

In an effort to evaluate the apoptotic cascades in dependency of iASPP we set up a proteome profiler human apoptosis array covering 35 proteins involved in apoptosis control. MOLM14_*iASPP* KD cell strains and the respective EV controls were treated with daunorubicin (100 nM, 30 h) and changes in protein expression were assessed accordingly (heat map provided with Fig. 5A).

Notably, upon daunorubicin treatment, *iASPP*-interference lead to restoration of p53 activation in this *TP53* wt model as indicated by phosphorylation of important p53 phospho-sites, such as S46, which is crucial for induction of intrinsic apoptosis [26]. Consequently, daunorubicin treatment resulted in increased expression levels of BAD, BAX and cleaved caspase 3 in the *iASPP* KD cell strains when compared to the EV controls.

In addition, silencing of *iASPP* lead to an increase of p27, pointing to reinstated cell cycle control, which may explain attenuation of cellular proliferation in the respective *iASPP* KD cells. Interestingly, we found catalases and receptors of TNF-related apoptosis-inducing ligands (TRAIL-R), known trigger of extrinsic apoptosis, upregulated in the *iASPP* KD strains, suggesting a broader and also p53-independent role of iASPP in controlling apoptosis induction and cellular homeostasis (Fig. 5B).

In this context, we established a second *iASPP* KD model, using the acute myeloid leukemia cell line HL60, which has a *TP53* null (-17,-17) background (DMSZ.de). Interrestingly, iASPP-interference again resulted in attenuation of cellular proliferation rates and sensitization towards chemotherapy in the respective HL60 *iASPP* KD cells, when compared to the EV strains. Treatment with daunorubicin or cytarabine thereby revealed significantly higher apoptosis rates in the *iASPP* KD cells, which is also reflected by significantly lower respective IC50s for daunorubicin and cytarabine in the *iASPP-*interferenced compared to the EV control strains (Supplementary Fig. S5). Together, these data support the notion of additional p53-independent functions of iASPP.

### iASPP drives leukemia biology in an in vivo mouse model

**Fig. 6:**
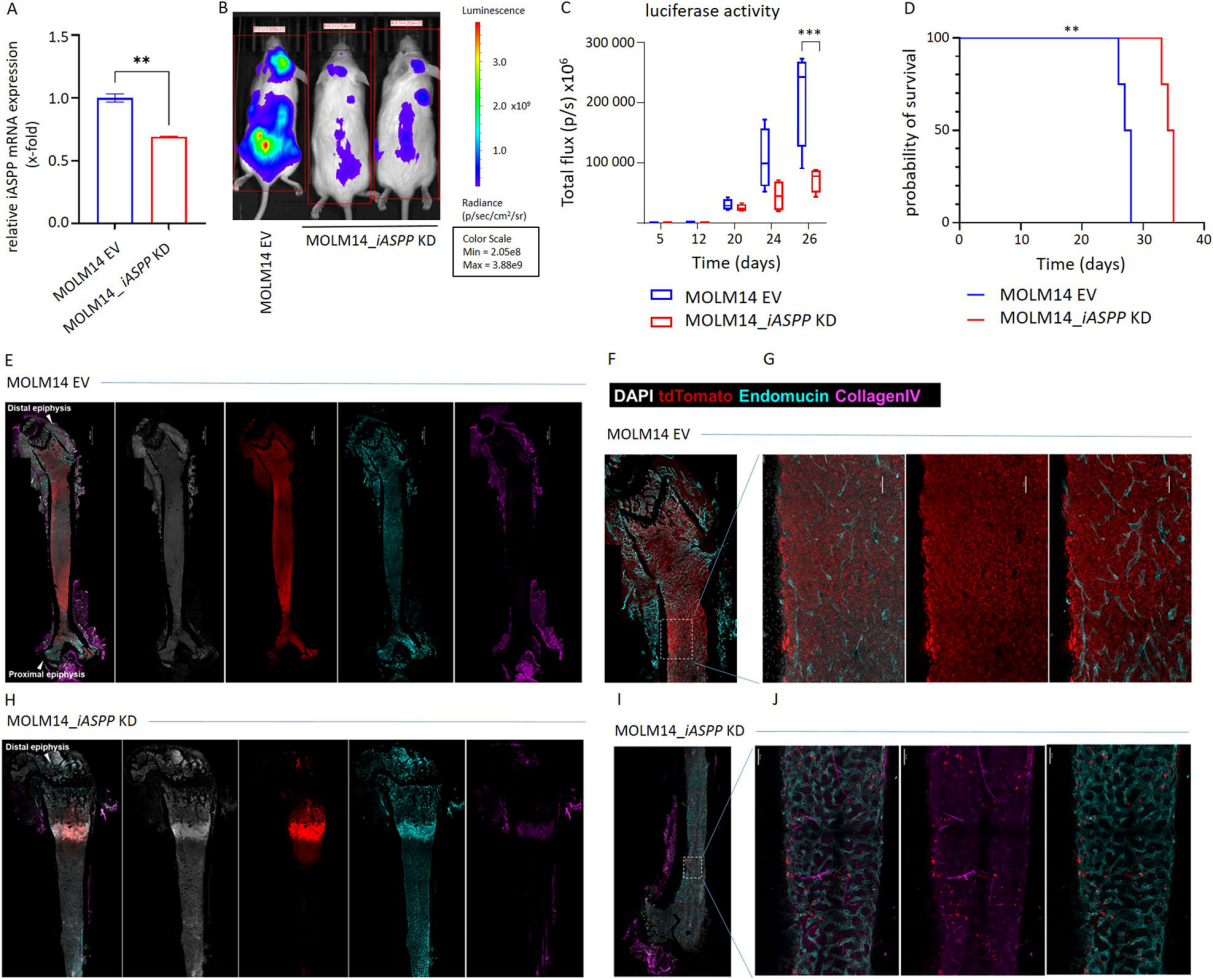
Xenotransplant mouse model according to iASSP expression. **A** Transduction efficiency assessed by mRNA. **B** In vivo visualization of luciferase activity in MOLM14_*iASPP* KD_Luc+ vs. MOLM14_EV_Luc+ xenotransplanted mice on day 26 post transplantationem. 3 representative mice shown. **C** Luciferase activity over time. **D** Survival rates over time (*n* = 4 EV / 4 KD). **E** Whole femur of a representative MOLM14 EV control mouse, stained with DAPI, tdTomato, Endomucin, aSMA and CollagenIV, 10x magnification. **F**, **G** Zoomed-in images of BM regions that were densely infiltrated by MOLM14 cells. (**H**) MOLM14-*iASPP* KD Luc+ mouse femur with a single MOLM14 infiltration zone under the growth plate of the distal epiphysis and (**I**, **J**) zoomed-in image of the proximal epiphysis. **p ≤ 0.01 (Log-Rank Mantel Cox test), ***p ≤ 0.001 (unpaired t-test).

To assess whether the in vitro observations with regard to altered proliferation rates in *iASPP*-interferenced leukemia cells translate into reduced tumor engraftment and disease progression in vivo, a MOLM14_*iASPP* KD_Luc^+^ xenotransplant NSG mouse model was established: 6 weeks old mice were injected IV with stably *iASPP*-interferenced cell strains or the EV mock controls. Leukemia engraftment and spreading were monitored regularly by checking white blood cell (WBC) counts, wellbeing and weight of the animals, as well as by employing a luciferase-based IVIS in vivo imaging system. Transfection efficiency is shown in Fig. 6A. Luc^+^ cells were sorted to maintain >85% enrichment. The methodology is described in more detail in the methods section.

Two separate approaches with respect to the endpoint of the experiment were followed: the primary endpoint of the pilot mouse experiment (*n* = 9) was overall survival, the confirmation experiment (*n* = 15) aimed to assess disease burden on a defined day (d28 post transplantationem).

Engraftment was confirmed in both, the *iASPP*_KD (transfection efficiency confirmed in Fig. 6A) and the EV cohorts by bioluminescence imaging. Importantly, disease progression and generalization was significantly attenuated in the *iASPP*_KD cohort (Fig. 6B, C) and this resulted in longer survival probabilities (Fig. 6D) when compared to the control mice (the entire population assessed over time is provided with Supplementary Figs. S6 and S7).

Of note, mice xenotransplanted with the *iASPP-*expressing mock strains presented with a quickly progressing emaciated phenotype, going along with a significant relative weight loss (Supplementary Fig. S8A). Consequently, the femur of a representative MOLM14 EV control mouse demonstrated dense infiltration of bone marrow with MOLM14 leukemia cells, recognizable by their larger nuclei and a dimmer DAPI signal. As a result, the stromal components (arteries [Collagen IV+ and aSMA +], sinusoids [Endomucin +] and extracellular matrix [Collagen IV +]) were perturbed in regions infiltrated by MOLM14 cells, with sinusoidal vessels displaying an apparently lower density and contracted lumen (Fig. 6E–G).

In contrast, a representative femur of a MOLM14_*iASPP* KD Luc+ transplanted mouse revealed much lower MOLM14 infiltration with a single cluster under the growth plate of the distal epiphysis. This area shows a more primitive but still present change in morphology of the sinusoidal vasculature with very thin vessels that infiltrate the tumor mass. In the proximal epiphysis, MOLM14 cells are only scattered, some of which seem to be localized along arteries (Collagen IV +) (Fig. 6H).

Interestingly, leukocyte counts in the peripheral blood did not differ between the EV and the *iASPP*-interference strains (Supplementary Fig. S8B) – instead, in vivo imaging did reveal high leukemic infiltration of the neurocranium (Supplementary Fig. S8C, D).

In the validation experiment all mice were sacrificed on day 28 to assess for tumor burden. Employing IVIS Lumina imaging, a higher degree of leukemic infiltration of brain, liver, spleen, femur and lungs was found in mice xenotransplanted with the MOLM14 EV strains in direct comparison to the *iASPP*_KD strains (Supplementary Fig. S9), which is in line with the reduced fitness and survival rates of EV mice in the pilot group.

## Discussion

Despite significant efforts to improve survival outcomes, acute myeloid leukemia remains a disease with a high mortality rate. Curative therapy typically involves intensive remission induction chemotherapy followed by repetitive cycles of consolidative chemotherapy or, depending on the prognostic risk profile, stem cell transplantation [1]. There are several prognostic risk scores available, such as those from the European LeukemiaNet [1] or NCCN (NCCN.org, V3.2023) [2]. However, relapse rates remain high - even among patients in the so-called favourable risk group, who still have a significant risk of initial treatment failure or disease relapse. Therefore, additional predictive markers are needed to more accurately identify patients at risk of treatment failure.

We here provide evidence that iASPP, a direct inhibitor of p53, is frequently overexpressed in AML and associates with a dismal patient outcome. This observation was confirmed in independent patient cohorts deriving from a large RNAseq data platform as well as an additional smaller random cohort of newly diagnosed AML from our center. The data is thereby in line with a previous report evaluating *iASPP* mRNA expression in acute leukemia, which included AML [19].

Importantly, we show that *iASPP* translates into a genuine protein isoform, which points to a functional role of iASPP in AML. Overexpression of iASPP was confirmed in the leukemia samples when compared to mononuclear cells from bone marrow donors. However, individual expression patterns varied widely and were not specific to bone marrow v*s*. peripheral blood samples, diagnostic qualifiers, de novo or R/R AML or ELN risk groups. Subcohort studies were limited by the small number of patient samples available – but still provided a similar pattern with a trend to higher mean expression levels in leukemias with a more aggressive biology. Higher iASPP expression was specifically found in secondary AML and refractory/relapsing AML as well as higher risk leukemias according to the ELN 2017 risk classification and AML failing induction chemotherapy. These observations suggest that iASPP may provide additional prognostic information.

We also screened a large transcriptomic database of a well-defined patient cohort with newly diagnosed AML and clinical follow-up data [22] to investigate the predictive value of iASPP in AML. Our findings indicate that high iASPP expression in this population is associated with unfavorable survival rates with regard to OS and EFS, respectively.

These findings on survival were confirmed using a validation cohort from a clinical trial population of patients treated with intensive chemotherapy protocols (HOVON102) [24]. In line, these results reveal profound significant differences in OS and EFS. Notably, high levels of *iASPP* predicted for an early event within the first year, again arguing for a biological role of iASPP as a resistance mechanism towards chemotherapy.

We next determined whether expression levels of *iASPP* reflect current risk stratification scores. Patients were classified according to cytogenetic and genetic risk groups according to ELN, and *iASPP* expression levels were assessed in these cohorts. Intriguingly, high *iASPP* levels predicted for an adverse outcome in all tested molecular categories and patient cohorts. Importantly, *iASPP* expression levels were not reflected by the ELN 2017 nor the ELN 2022 risk classification but added additional information to identify patients with a higher likelihood for a disadvantageous outcome in all risk categories. This information allows a more accurate prediction of survival outcomes within the ELN categories and may have direct therapeutic implications, such as the choice of consolidation therapy in favorable and intermediate risk patients, where patients with highly expressing *iASPP* may benefit from a more intensive treatment strategy, including consolidating allogeneic stem cell transplantation. Therefore, determining *iASPP* expression would provide important additional predictive information to guide therapy decisions.

An attempt to assess the significance of *iASPP* expression patterns in genetic subgroups uniformly confirmed the in-principle adverse effect of high *iASPP* expression levels, whereas significance was not met due to small patient numbers. Of interest, overexpression of *iASPP* trended to associate with an adverse outcome in patients harboring *TP53* mutations – which is remarkable, as iASPP is a direct binding partner of p53 to inhibit p53-mediated apoptosis [13, 27]. This observation argues for additional p53-independent functions of *iASPP* in acute myeloid leukemia.

To assess whether the clinical observations linked to high iASPP expression directly translate into genuine functional consequences, several leukemia *iASPP*-interference models were generated for in vitro, ex vivo and in vivo assessment, including patient leukemic blasts as well as a *TP53* wt (MOLM14) and a *TP53* null (HL60) cell line model.

All experimental setups confirmed that iASPP directly contributes to a more aggressive tumor biology – and silencing of *iASPP* attenuates the proliferative capacity of leukemic blasts. Notably, decreased cellular proliferation rates were observed in cells with a *TP53* wt background as well as in the *TP53* null cell model, arguing for additional p53-independent functions of iASPP. This observation is in line with findings reported by Liu and colleagues in *TP53*-defective bladder cancer [28] – whereas others have demonstrated p53-dependent mechanisms in non-small cell lung cancer via direct regulation of p21 and PUMA [29].

Importantly, the functional role of iASPP in leukemia biology was further confirmed in vivo: NSG mice xenotransplanted with an *iASPP*-interference MOLM14 leukemia cell model revealed a significantly earlier disease onset, dissemination and progression of acute leukemia of the EV strains as evidenced by phenotype and IVIS Lumina imaging. These observations translated into a significantly shorter overall survival and intense leukemic organ infiltration of mice xenotransplanted with iASPP-expressing blasts, again strengthening our findings that high iASPP expression correlates with and predicts for an aggressive tumor phenotype and a dismal outcome in AML patients.

Still, the best known function of iASPP is the (inhibitory) control of apoptosis induction via direct interaction with p53 [13]. This is in line with our observation in the clinical studies, which consistently demonstrated an adverse effect of high iASPP expression on therapy response and clinical outcome in all cohorts and subcohorts studied. We therefore employed our *iASPP*-interference cell models to assess for apoptosis induction in response to (chemo)therapy – and confirm restoration of apoptotis upon attenuation of iASPP expression. Importantly, re-sensitization towards chemotherapy upon *iASPP* interference was observed in an ex vivo model using freshly harvested leukemic blasts as well as in the MOLM14 *TP53* wt and in the HL60 *TP53* null cell lines, again arguing for – at least in part – p53-independent functions of iASPP. In line, using the MOLM14 *iASPP* KD model, proteome pathway analyses revealed that attenuation of iASPP results in activation of p53-dependent, intrinsic as well as p53-independent, extrinsic apoptosis signaling. These observations underline the central functional role of iASPP as an independent checkpoint to control induction of apoptosis.

Since iASPP belongs to a family of p53-binding proteins, it is intriguing to hypothesize that other members of the ASPP family (i.e. proapoptotic ASPP1 and ASPP2, with binding capacities to p53 but antagonistic effects with regard to induction of apoptosis) may have an impact on therapy resistance and survival outcomes as well. Indeed, we have shown in previous works [9, 10] that attenuation of ASPP1 (*PPP1R13B*, further ref. to as *ASPP1*) or ASPP2 (*TP53BP2*, further ref. to as *ASPP2*) results in therapy resistance in AML. We now confirm these observations in the transcriptomic dataset (GSE6891) described in Fig. 2 and performed a multivariate analysis simultaneously including *iASPP* as well as *ASPP1* and *ASPP2* to assess for expression levels in comparison to survival outcomes. Together and in line with our previous work, *ASPP1* barely failed significance (*p* = 0.80) as an independent predictor of survival outcome, whereas *iASPP* (*p* = 0.0002) and *ASPP2* (*p* = 0.02) proofed significance – with *iASPP* being the strongest predictor in this multivariate analysis (data not shown). We also addressed whether *ASPP1* and/or ASPP2 have a direct influence on the adverse survival outcomes seen for *iASPP*^high^ expressor cohorts. Tantalizingly, analysis of the cohort highly expressing iASPP reveals that co-expression of ASPP2 (*p* = 0.00004), *ASPP1* + *ASPP2* (*p* = 0.0053), but not *ASPP1* alone (*p* = 0.27) has a benefitial effect with regard to OS (i.e. compensates the adverse effect of *iASPP* in this high risk cohort, Supplementary Fig. S10). This observation raises the scope to the next level and detailed analyses of all ASPP family members in different patient populations and risk cohorts need to be addressed in future studies.

The cause for deregulated iASPP in cancer is unknown. For the pro-apoptotic ASPP family members, ASPP1 and ASPP2, methylation of the promoter region has been shown by us and others to repress transcription in leukemia [9, 10, 30]. For iASPP, putative binding sites of GATA-2, a transcription factor of multilineage hematopoiesis which is overexpressed and dysregulated in AML [31, 32], have been discussed in the promoter region of *iASPP* [33]. However, this observation needs confirmation and the precise regulation of iASPP is still unknown.

Of interest, CDK9 inhibitors were identified to reactivate p53 via downregulation of *iASPP* in a colon cancer model [34]. This observation provides a rationale to target *iASPP* overexpressing cancers with a CDK9 inhibitor to restore p53-mediated induction of apoptosis and sensitize tumor cells for cytotoxic drugs. CDK9 inhibitors, such as AZD4573 [35] are under clinical investigation in hematologic malignancies (ClinicalTrials.gov Identifier: NCT03263637). Another CDK9 inhibitor, SLS009 (formerly GFH009), just obtained orphan drug designation by the FDA, based on results from a phase I trial that showed high rates of blast reduction and durable complete remissions (NCT04588922). Intriguingly, we have preliminary data demonstrating that CDK9i, using the CDK9 inhibitor AZD4573, results in a downregulation of *iASPP* in our MOLM14 cell model (Supplementary Fig. S11), which argues for a novel strategy to override iASPP-driven therapy failures. This strategy should be taken into consideration in future trials.

To summarize, we show that iASPP is frequently overexpressed and functionally active in AML, leading to a more aggressive tumor biology and reduced susceptibility towards chemotherapy. Independent patient cohorts uniformly confirm the adverse effect of *iASPP* with respect to survival probabilities. Importantly, the predictive value of *iASPP* is thereby not reflected by current risk classifications. Determination of iASPP expression levels provides valuable additional information as a predictive marker in AML and may guide treatment decisions.

## Methods and Materials

### Clinical patient cohorts

#### RNAseq cohort

A robust and comprehensive RNAseq dataset of an integrated database was compiled. In particular, these sources include three key repositories: the Cancer Genome Atlas (TCGA, https://www.cancer.gov/ccg/research/genome-sequencing/tcga), the Therapeutically Applicable Research to Generate Effective Treatments (TARGET, https://www.cancer.gov/ccg/research/genome-sequencing/target) database, and the Genotype-Tissue Expression (GTEx, https://gtexportal.org/) database of normal tissue. The combination of the three databases creates a large and comprehensive dataset comprising *n* = 56,938 samples [20].

#### Expression validation cohort

Bone marrow aspirate and peripheral blood samples from 99 patients with diagnosed AML (patient characteristics are provided in Supplementary Table S1) and 31 healthy bone marrow volunteers were collected in 5000 U heparin after written informed consent and approval of the ethics committees of the University of Tübingen (project number 405/2006BO2) and Eastern Switzerland (EKOS 20/128). Mononuclear cells were isolated by Ficoll Hypaque density gradient fractionation [36].

#### Clinical training cohort

A transcriptome dataset from Gene Expression Omnibus (GEO) was used as a training population. This dataset includes two independent representative cohorts consisting of 247 and 214 patients with de novo AML, aged ≤60 years (www.ncbi.nlm.nih.gov/geo, accession number GSE6891) [22].

#### Clinical validation cohort

AML patients were intensively treated on consecutive Dutch-Belgian Hemato-Oncology Cooperative Group (HOVON) and Swiss Group for Clinical Cancer Research (SAKK) clinical protocol HO102 (18–65 yr) [22, 24]. The treatment protocol and patient eligibility criteria are available at http://www.hovon.nl.

### Antibodies and reagents

An anti-iASPP monoclonal mouse antibody (#A4605, Sigma), targeting an epitope within amino acids 691–1128, was used at a 1∶1000 to 1∶250 dilution. Fluorescent dye-conjugated (AlexaFluor®) secondary goat anti-mouse was used for flow cytometry studies according to standard protocols (#4408, Cell Signaling, MA/USA).

Daunorubicin, cytarabine and sunitinib were obtained from the University of Tübingen Hospital Pharmacy and AZD4573 was purchased (Selleckchem, Tx/USA) and dissolved in DMSO to create stock solutions and stored at −20 °C.

### Polymerase Chain Reaction (PCR)

mRNA was isolated and reversely transcribed using standard techniques and a RNeasy® RNA purification kit (Qiagen, Germany). *iASPP* expression levels, relative to *GAPDH* as the housekeeping gene, were determined by qRT-PCR Roche® LightCycler Technology (Roche, CH). Relative fold expression of the target gene transcript in comparison to the reference gene transcript was calculated using the 2–∆∆Ct method. The primers were set as follows: human *iASPP* 5‘-CCC CAT CAC TGA GGG ATC T-3‘ and 5‘-AGC ACAGAG CGC ATC TCC-3‘; human *GAPDH* 5‘-AGC CAC ATC GCT CAG ACA C-3‘ and 5’GCC CAA TAC GAC CAA ATC C-3‘.

### Flow cytometry-based iASPP protein expression

Cells were fixed and permeabilized using the Fix & Perm® Fixation and Permeabilization kit (ADG An der Grub Bioresearch, Austria). A non-specific IgG isotype control was used as a baseline and expression levels of the protein of interest and the respective controls were assessed using a FACScalibur® flow cytometer loaded with CellQuest® analysis software (BD, NJ/USA).

### Immunohistochemistry

Paraffin-embedded EDTA-decalcified bone marrow biopsies were used for immunohistochemistry assessment of iASPP protein expression as previously described [37]. Hematoxylin counterstains were used as negative controls. Slides were evaluated on a Zeiss® Axioscope 40 (Carl Zeiss, Germany).

### Cell lines

Mycoplasma-certified leukemia cell lines MOLM14 and HL60 were obtained from the German Collection of Microorganisms and Cell Cultures (Leipniz Institute DSMZ, Germany). Cells were cultured in RPMI 1640, supplemented with 10% fetal bovine serum, 1% penicillin G (10,000 units/mL), 1% sodium pyruvate, 1% NEAA, streptomycin (10,000 µg/mg) and 2 mmol/L l-glutamine (GIBCO/Thermo Fisher Sci, MA/USA).

### Lentiviral shRNA knock-down in human AML cell lines and leukemic blasts

A lentiviral short hairpin (sh)RNA interference model was established to create leukemia cell strains stably suppressing iASPP. A GIPZ lentiviral shRNAmir-based protocol was followed (Thermo Fisher Sci, MA/USA). In short, lentiviral vectors were cloned in HEK293T cells. Successful transduction was GFP-monitored and leukemia cells were puromycin-selected to establish stable cell strains. Empty vector (EV) cell strains were used as negative controls. Successful *iASPP*-interference was confirmed on the transcriptional level by qRT-PCR and on the protein level by flow cytometry using an anti-iASPP polyclonal rabbit antibody (#18590-1-AP, Proteintech, UK).

### Apoptosis assays

Induction of apoptosis was analyzed using an Annexin V-based flow cytometry assay (Immunotech/Beckman Coulter, France) as described previously [38]. Translocation of phosphatidylserine from the inner to the outer leaflet of the plasma membrane is thereby indicative for early apoptosis. A co-stain with propidium iodide is used to assess for late apoptotic and dead cells. All experiments were performed at least in triplicates.

### Proteom Profiler human apoptosis array

To evaluate the impact of iASPP knock-down in AML cells on a larger number of proteins involved in regulation of apoptosis, a proteome array was performed on MOLM14 EV and *iASPP* KD cells.

The Proteome Profiler™ Human Apoptosis Array Kit (R&D Systems Cat #ARY009, MN/USA), was used to characterize apoptosis-related protein profiles after treatment with 100 nM daunorubicin for 30 h according to the manufacturer’s instructions.

Briefly, proteins in total cell lysates were quantified and 400 μg of lysates were loaded per membrane and incubated overnight at 4 °C. The next day, the array membranes were incubated with a Detection Antibody Cocktail for 1 h and thereupon with Streptavidin-HRP for 30 minutes at room temperature. A Chemi Reagent Mix was applied for visualization of the array results. Membranes were scanned on a ChemiDoc Imaging System (Biorad, CA/USA) and protein expression was analyzed quantitatively using ImageJ v1.53k (National Institute of Health). After initial background subtraction, the mean gray value of every dot (protein) was measured independently using the same circular selection. Taking into account the negative controls and correction factors from the positive controls, a mean value was calculated from the duplicates for each protein and used for the expression analyses.

The heatmap was generated using GraphPad Prism 9.4.1.

### Cell viability and proliferation assays

Cleavage of tetrazolium salts (XTT) was measured at certain time points according to the manufacturer’s instructions (Abcam, UK), to determine the relative quantification of viable, proliferating cells. The measured absorption rates thereby correlate with the number of metabolically active cells. In addition, results were confirmed by assessing cell doubling times. To determine the proliferation capacity, cell numbers were subsequently counted for 4 days at fixed 24 hour intervals using a Neubauer Counting Chamber. Assuming an exponential growth, formula 1 was used to determine the growth constant λ. Cell doubling time (t_d_) was determined using formula 2. All experiments were performed at least in triplicates.

$$\begin{array}{cc}{\rm{Formula}}\,1 & {\rm{Formula}}\,2\\ \lambda =\frac{{\rm{In}}({{N}}_{{t}})-{\rm{In}}({{N}}_{0})}{t-{t}_{0}} & {t}_{d}=\frac{{\rm{In}}(2)}{\lambda }\end{array}$$

t_0_ = initial time, t = observation time, N_0_ = initial cell count, N_t_ = cell number at time t, λ = growth constant, t_d_ = doubling time.

### iASPP-interference xenotransplant mouse model

Homozygous male and female non-obese diabetic severe combined immunodeficiency gammac(-/-) (NOD *SCID* gamma (NSG)) mice were obtained from Charles River Laboratories International, Inc. (MA/USA) and housed in individually ventilated cages under conventional specific pathogen-free conditions, maintaining a 12 h light–dark cycle, 22 °C ambient temperature and 45–50% humidity. The experimental procedures were carried out in accordance with Swiss federal and cantonal guidelines (Tierschutzgesetz), granted by the Veterinary Office of the Canton of St. Gallen (SG09/2022).

Stably transfected MOLM14 *iASPP*-knock down (KD) or EV cell strains were cotransfected with luciferase pCDH-EF1-Luc2-P2A-tdTomato lentiviral particles (Sigma Aldrich, MO/USA) to enable in vivo imaging of disease development. TdTomato^+^ cells were sorted (enrichment > 85%) using a BD FACSMelody^TM^ Cell Sorter (BD, NJ/USA).

These cell strains were xenotransplanted IV into a tail vein of mice at the age of 6 weeks and followed for leukemia engraftment and disease progression: after 5 days, mice were subsequently injected IP with 1.5 mg/10 g D-luciferin solution (GlpBio, CA/USA). In vivo imaging was performed under isoflurane anesthesia using an IVIS Lumina III In Vivo Imaging System (PerkinElmer, MA/USA). Luciferase activity was evaluated as the total flux per mouse (p/s). The procedures were repeated weekly to monitor leukemia development and progression.

At the time of disease symptoms onset, mice underwent carbon dioxide euthanasia, according to the experimental scoresheets. Mouse organs were harvested and examined for tumor infiltration using the IVIS Lumina III In Vivo Imaging System (PerkinElmer, MA/USA), then snap-frozen for follow-up evaluations.

### BM slice preparation, immunostaining, and optical clearing

Mouse femurs were isolated, cleaned from the surrounding connective tissue, and fixed in 2% paraformaldehyde in PBS overnight at 4 °C. The samples were then dehydrated in 30% sucrose for 72 h at 4 °C to prevent ice crystals formation. Femurs were then embedded in cryopreserving medium (optimal cutting temperature (OCT)) and snap-frozen in liquid nitrogen. Bone specimens were sectioned using a cryostat until the BM cavity was fully exposed along the longitudinal axis on both sides. Once BM slices were generated, the remaining OCT medium was removed by incubating and washing the samples in PBS. For immunostaining, slices were incubated in blocking solution (0.2% Triton X-100, 10% donkey serum in PBS) overnight at 4 °C. Primary and secondary antibody incubations were performed in blocking solution for three days at 4 °C. Last, immunostained thick femoral slices were incubated in RapiClear 1.52 (SunJin Lab, Taiwan), for a minimum of 6 hours, to increase imaging depth without loss of signal intensity.

### Confocal imaging and Image processing

BM slices were mounted on glass slides using high-vacuum silicone grease. Confocal microscopy was performed with 10x and 20x objectives on a Stellaris 5 (Leica Microsystems, Germany) confocal microscope. Combinations of up to five dyes (DAPI, AF488, AF546, AF594, and AF680) were simultaneously used in the experiments, and their emitted fluorescence was detected using ultrasensitive Leica Hybrid detectors (HyD). Attenuation of fluorescent signal with tissue depth was minimized using the z-compensation tool available on the Leica Application Suite X software. Confocal image stacks were rendered into 3D volumes and analyzed using Imaris v10.0.0 (Bitplane, CH).

### Statistical analysis

#### RNAseq cohort

mRNA expression analysis was performed using the TNM Plotter web platform, a powerful computational tool available at TNMplot.com.

#### Expression validation cohort

In vitro data analysis was based on the unpaired two sample t-test using GraphPad Prism software. In presence of non-Gaussian distributed data, comparisons were performed using the nonparametric Mann-Whitney-U-Test.

#### Clinical training cohort

The raw data (CEL files) were downloaded for all patient samples, and the expression files were MAS5 normalized. Then, a scaling normalization was performed to set the mean expression to 1000 in each sample. The probe set with the ID 218849_s_at was used to determine iASPP expression levels. Survival analysis was performed by analyzing all available cutoff values between the lower and upper quartiles of expression and computing the false discovery rate to correct for multiple hypothesis testing. The survival plots at the best cut-off were visualized using the Kaplan-Meier method with the estimated HR values and 95% confidence intervals.

#### Clinical validation cohort

Cumulative overall survival (OS) and event-free survival (EFS) probabilities were estimated using the Kaplan-Meier method. Survival probabilities are reported as the percentage of patients surviving for 1 or 2 years, and median survival times are also provided. The log-rank or Wilcoxon test was used to test the difference between survival curves. Multifactorial analysis was performed using Cox regression analysis. Additionally, estimated hazard ratios (HR) with a 95% confidence interval are provided. These analyses were done using JMP 16.2 statistical software (SAS Institute, NC/USA).

#### Mouse model

Based on empirical values a non-randomized non-blinded mouse model was established using a pilot cohort (*n* = 4 + 5) and a validation cohort (*n* = 7 + 8).

## Supplementary information

Supplementary Material

## Data Availability

The materials described in the manuscript will be freely available to any researcher upon reasonalble request for the use of non-commercial purposes.
