# Supplementary material for: High iASPP (PPP1R13L) expression is an independent predictor of adverse clinical outcome in acute myeloid leukemia (AML)
Source: Cell Death Dis. 2024 Nov 30;15(11):869. doi: 10.1038/s41419-024-07190-8 (PMC11608330; doi:10.1038/s41419-024-07190-8)
Supplement: Supplementary file 1 — Supplementary Material [file 41419_2024_7190_MOESM1_ESM.pdf]

SUPPLEMENTAL INFORMATION

Supplemental Figure S1

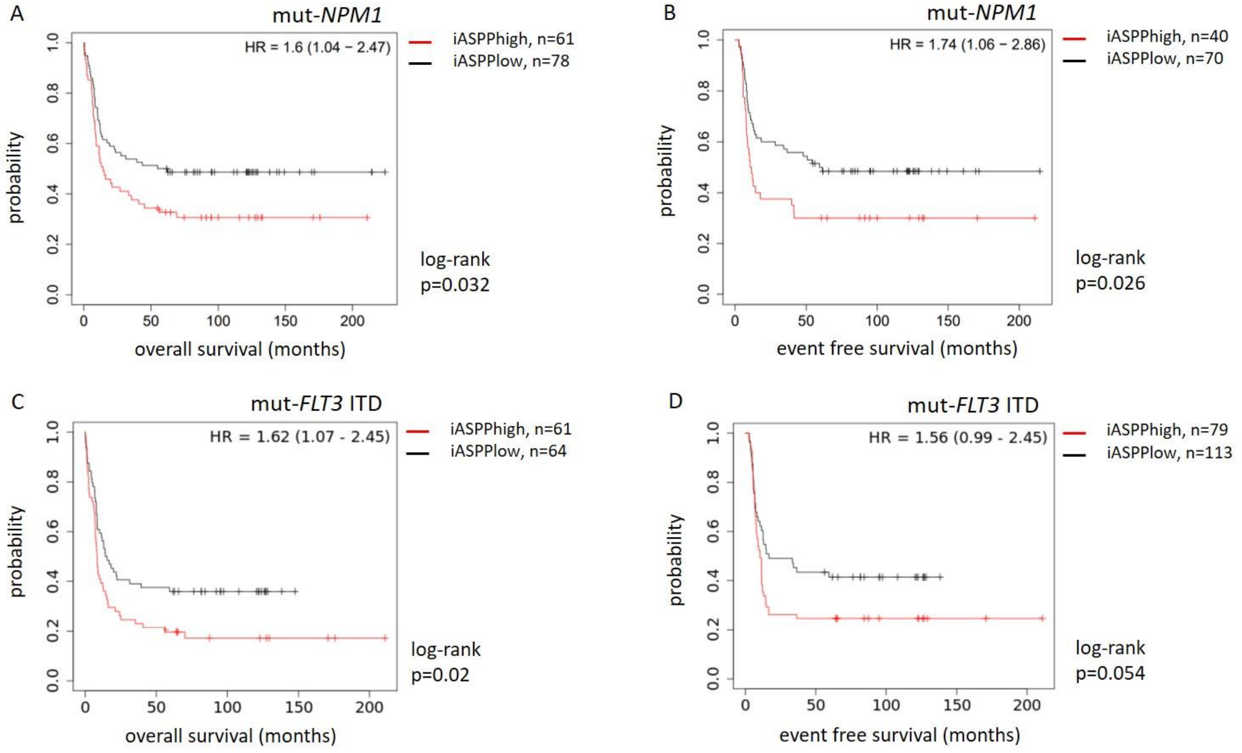

Supplemental Figure S1. iASPP and survival according to gene mutation. (A, C) OS and (B, D) EFS according to iASPP and gene mutation as assessed in a transcriptomic dataset (GSE6891).

Supplemental Figure S2

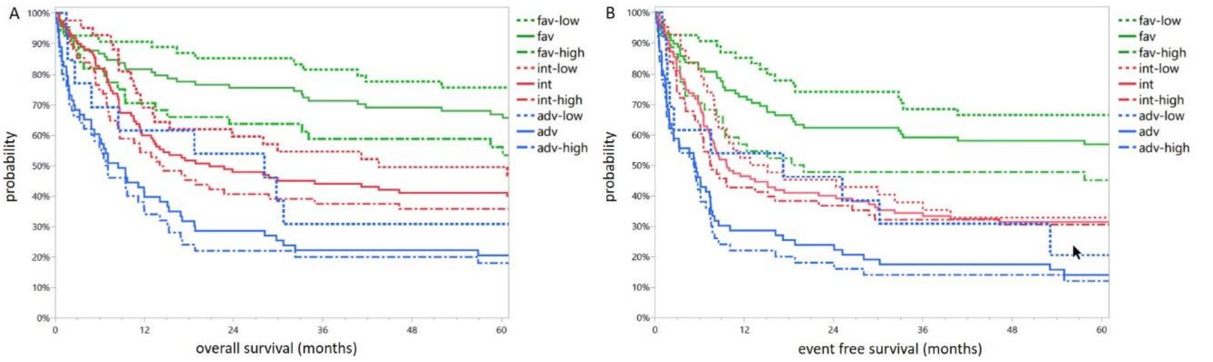

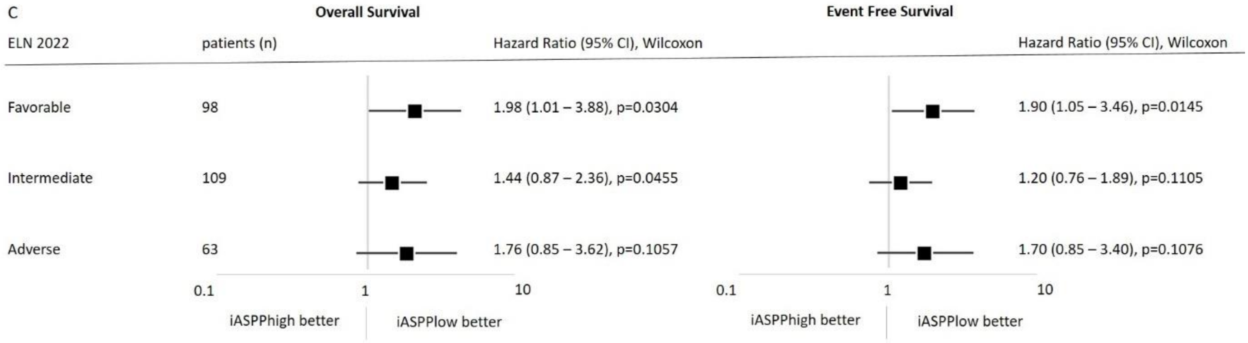

D

ELN 2022	OS	Overall Survival: iASPP high (95% CI)	Overall Survival: iASPP low (95% CI)	Event Free Survival: iASPP high (95% CI)	Event Free Survival: iASPP low (95% CI)
Favorable	median	NR (15.11-NR)	NR	19.2 mo (8.3-NR)	111.2 mo (61.6-111.2)
Intermediate	median	14.1 mo (8.6-34.9)	43.5 mo (13.4-NR)	7.5 mo (6.11-16.16)	14.0 mo (8.18-36.01)
Adverse	median	6.9 mo (3.28-11.96)	28.2 mo (2.66-NR)	5.2 mo (1.87-6.87)	17.26 mo (1.71-53.09)

Supplemental Figure S2. Probability of survival according to iASPP expression and ELN 2022 risk classification in a patient cohort treated in the HOVON102 trial (n=274). (A) OS, (B) EFS, (C) HR and (D) median OS provided.

Supplemental Figure S3

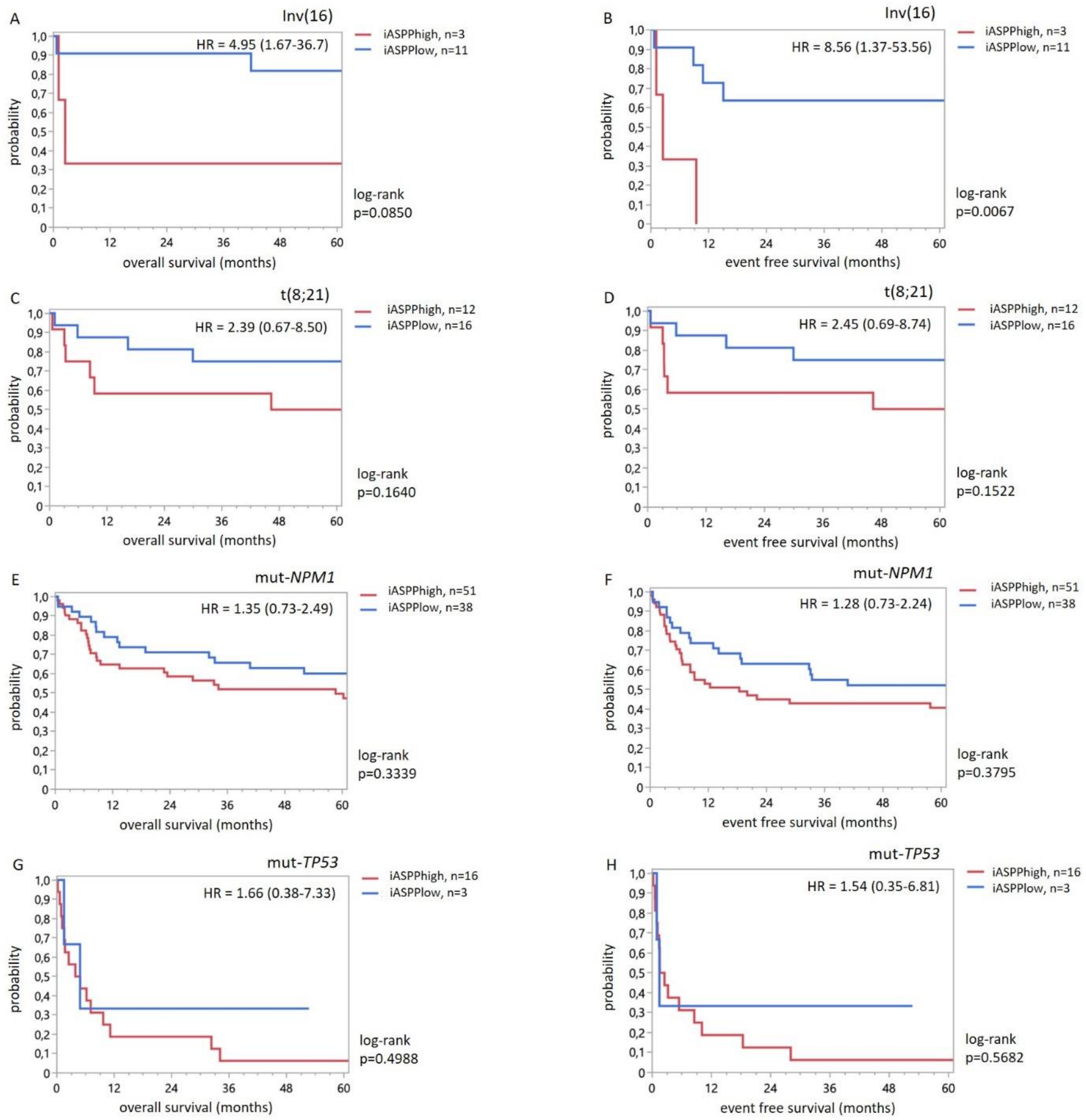

Supplemental Figure S3. iASPP and survival according to gene mutation. (A, C, E, G) OS and (B, D, F, H) EFS according to *iASPP* expression levels and gene mutation status in a patient set treated in the HOVON102 trial.

Supplemental Figure S4

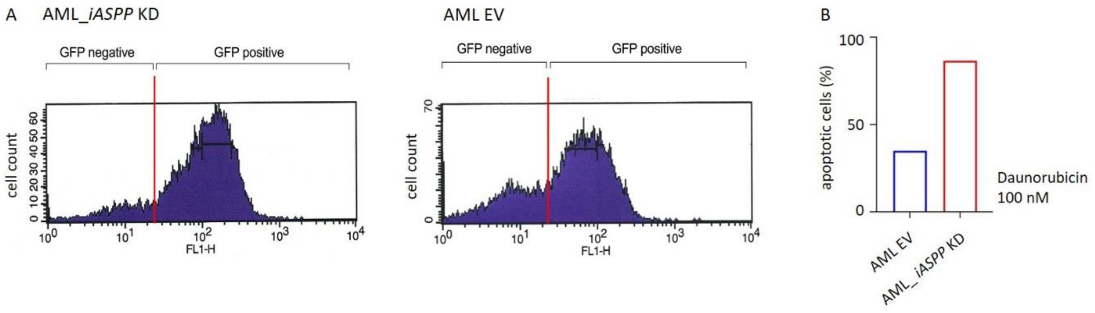

Supplemental Figure S4. Cytoreductive capacity after *iASPP*-interference in freshly isolated native leukemia blasts *ex vivo*. (A) Transduction efficiency as monitored by fluorescent GFP signal detection. (B) Cytoreduction after treatment with daunorubicin (100nM) in empty vector (EV) and iASPP knock down (KD) cell strains.

Supplemental Figure S5

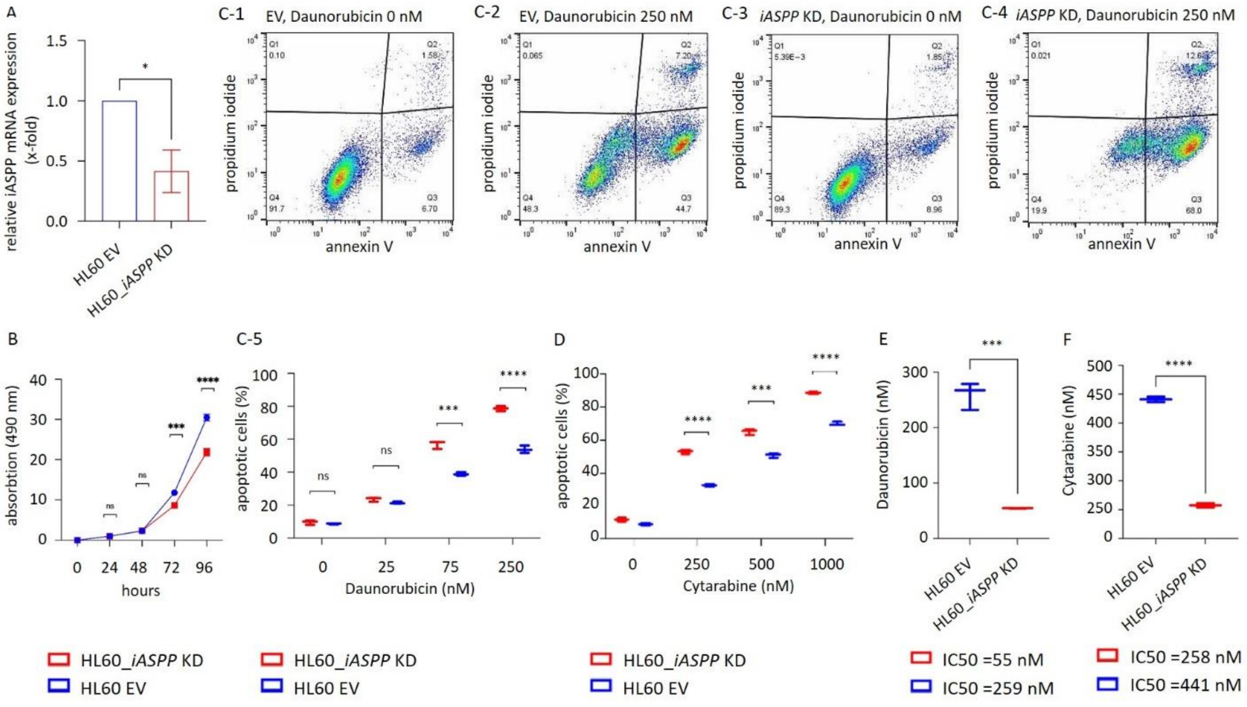

Supplemental Figure S5. Functional analysis of iASPP in a HL60 interference cell line model. (A) Transduction efficiency assessed by mRNA. (B) *iASPP*-interference results in reduced cellular proliferation, indicated by reduced metabolic activity as assessed by XTT. (C) Induction of apoptosis in response to daunorubicin as measured in an Annexin V/PI flow cytometry approach (C1-4 exemplary dot plots shown, C-5 dose-dilution graph of the entire experiment. (D) Induction of apoptosis in response to cytarabine. (E/F) Box plot analysis to determine IC_{50} concentrations compared to empty vector (EV) control strains. Technical triplicates minimum for all assays. * $p \leq 0.1$, ** $p \leq 0.01$, ≤ *** $p \leq 0.001$, **** $p \leq 0.0001$ (t-test)

Supplemental Figure S6

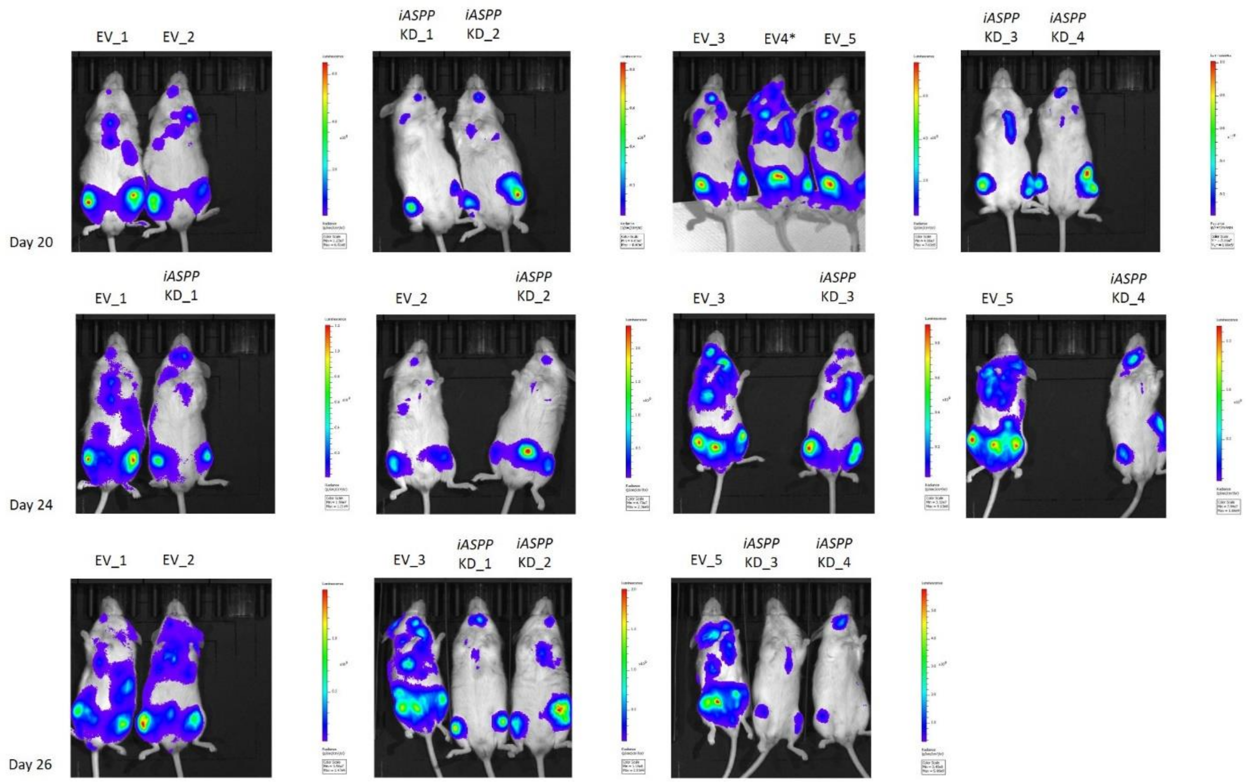

Supplemental Figure S6 (pilot cohort, n=9). Xenotransplant mouse model according to iASSP expression. In vivo visualization of relative intensities of luciferase activity in MOLM14_iASPP KD_Luc+ vs. MOLM14_EV_Luc+ xenotransplanted mice on days 20, 24 and 26 post transplantationem. Entire population shown (n=9), ventral view

Supplemental Figure S7

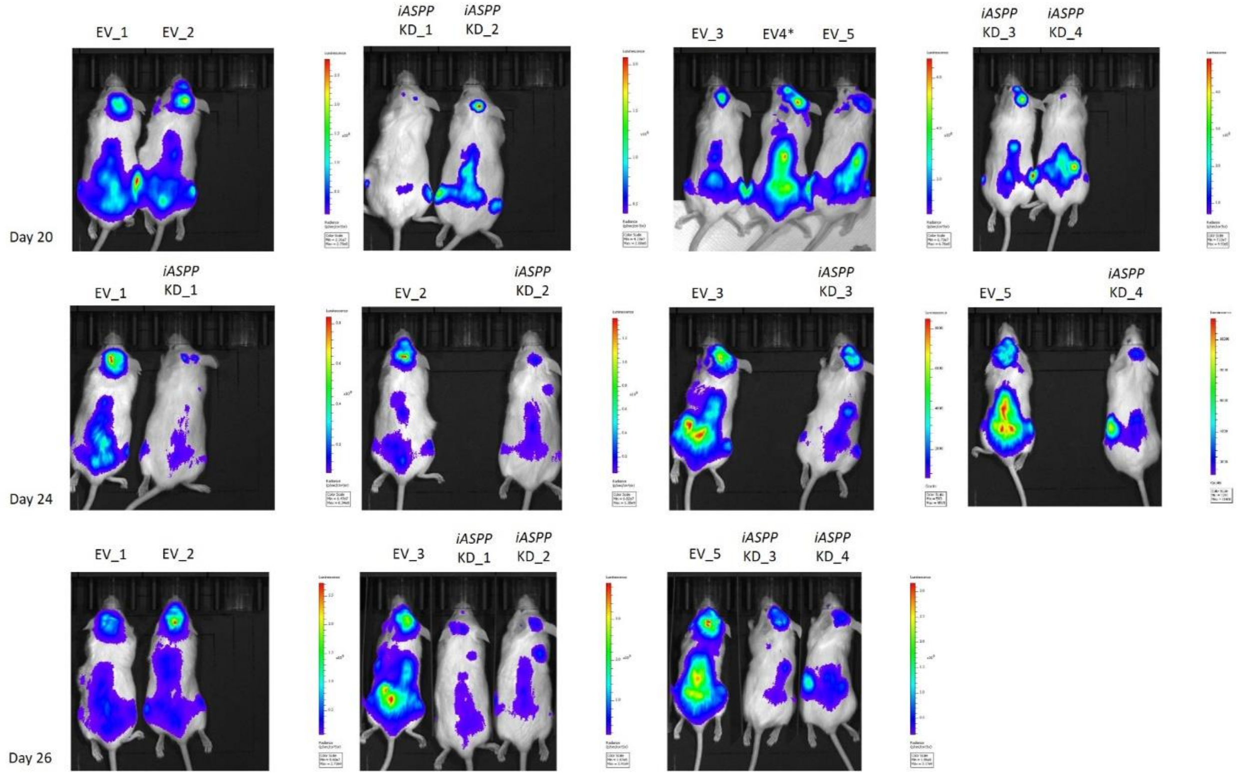

Supplemental Figure S7 (pilot cohort). Xenotransplant mouse model according to iASSP expression. In vivo visualization of relative intensities of luciferase activity in MOLM14_iASPP KD_Luc+ vs. MOLM14_EV_Luc+ xenotransplanted mice on days 20, 24 and 26 post transplantationem. Entire population shown (n=9), dorsal view

Supplemental Figure S8

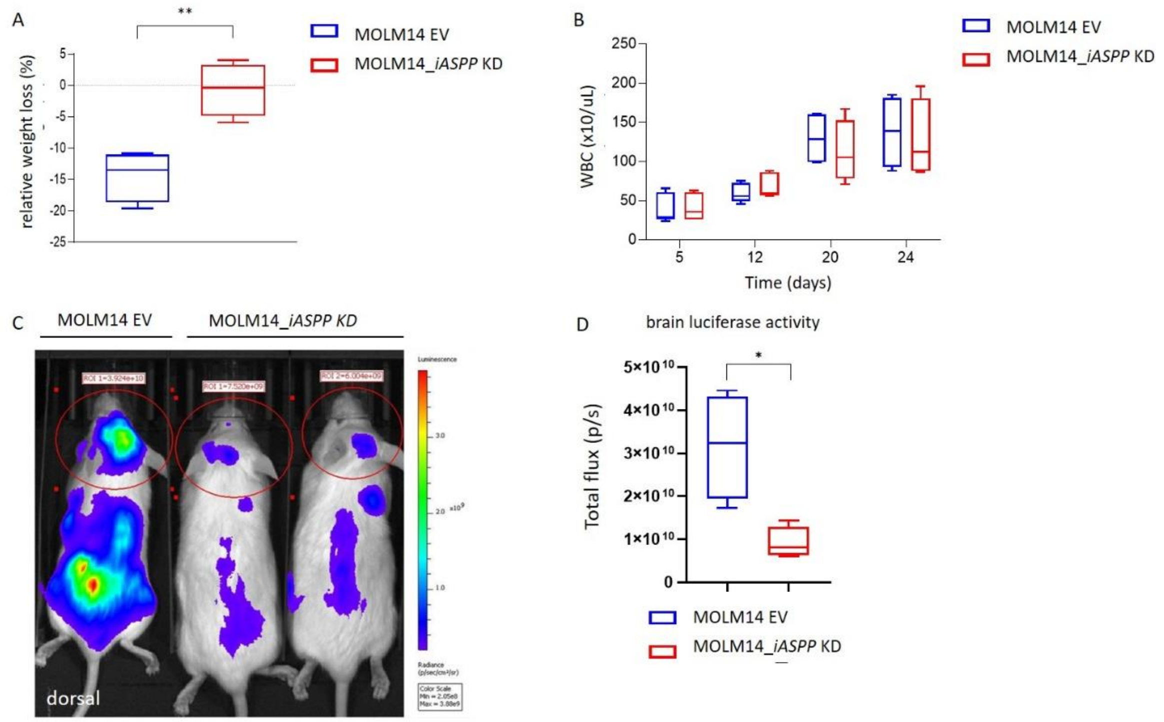

Supplemental Figure S8 (pilot cohort). Xenotransplant mouse model according to iASSP expression. (A) Relative weight loss in percent of the initial weight (n=9). (B) White blood cell count, EV: n=5, KD: n=4. (C) In vivo bioluminescence of luciferase activity with a focus on neurocranial infiltration in MOLM14_iASPP KD_Luc+ vs. MOLM14_EV_Luc+ xenotransplanted mice on day 26 post transplantationem. 3 representative mice shown. (D) Brain luciferase activity on day 26 post transplantationem. * p ≤ 0.05, ** p ≤ 0.01 (unpaired t-test)

Supplemental Figure S9

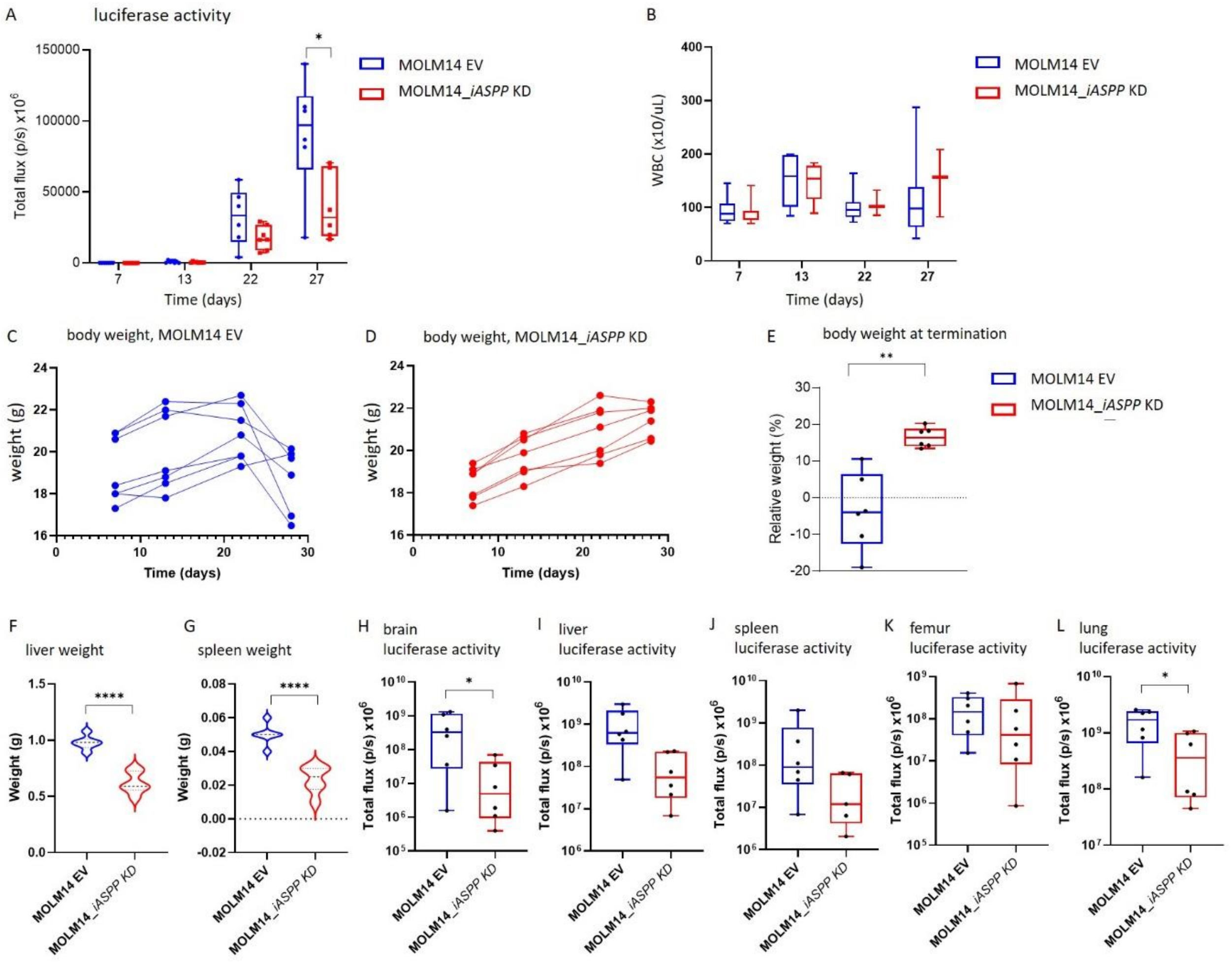

Supplemental Figure S9 (validation cohort). Xenotransplant mouse model according to iASSP expression. (A) Absolute luciferase activity in MOLM14_iASPP KD_Luc$^+$ *vs.* MOLM14_EV_Luc$^+$ xenotransplanted mice over time, n=15. (B) White blood cell count. (C-E,) Relative weight loss in percent of the initial weight. **p ≤ 0.001 (unpaired t-test) (F-G) Weight of liver and spleen. (H-L) Luciferase activity of brain, liver, spleen, femur and lung. * p = 0.036 (unpaired t-test); (C-L: n=12 mice available for final analyses)

Supplemental Figure S10

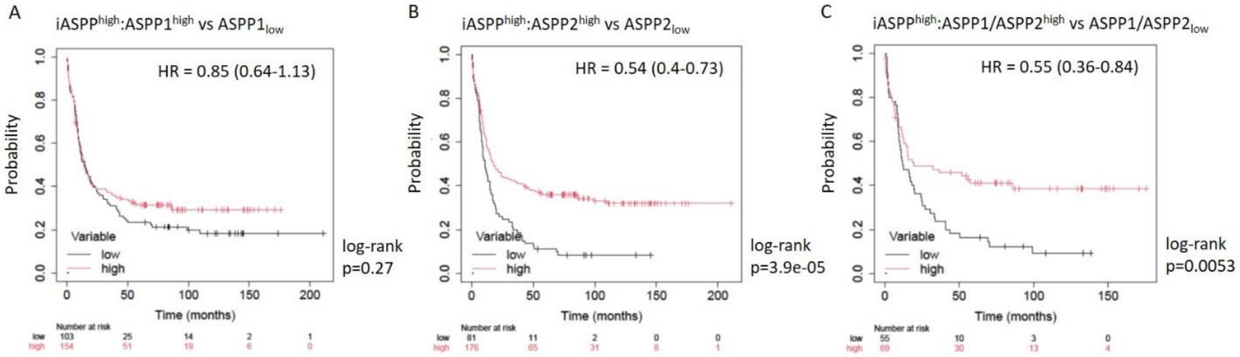

Supplemental Figure S10. Overall survival according to ASPP1 and/or ASPP2 coexpression with iASPP. Analysis of a iASPP high-expressor cohort (transcriptomic dataset (GSE6891)) reveals that coexpression (variable) of ASPP2 (p=0.00004), ASPP1+ASPP2 (p=0.0053), but not ASPP1 (p=0.27) has a beneficial effect with regard to OS (i.e. compensates the adverse effect of iASPP in this cohort).

Supplemental Figure S11

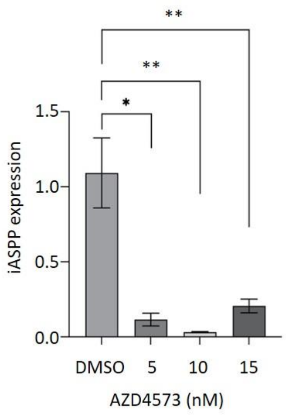

Supplemental Figure S11. *iASPP* expression after CDK9 inhibition. AZD4573 treatment results in significantly reduced iASPP expression in the MOLM-14 cell line compared to DMSO-treated control cells as determined bei RT-qPCR. Three independent measurements shown. * $p \leq 0.05$, ** $p \leq 0.01$ (t-test)

Supplemental Table S1. Clinical characteristics of an unselected training cohort (figure 1)

Spec.	ELN	AML qualifier	Analysis	Spec.	ELN	AML qualifier	Analysis	Spec.	ELN	AML qualifier	Analysis
P1	int	*de novo*	Fc	P34	adv	*de novo*	Fc, mRNA	P67	fav	R/R AML	Fc, mRNA
P2	fav	*de novo*	Fc	P35	adv	sAML	Fc	P68	fav	sAML	Fc
P3	adv	*de novo*	Fc	P36	adv	*de novo*	Fc, mRNA	P69	int	R/R AML	Fc, mRNA
P4	n.d.	n.d.	Fc	P37	adv	sAML	Fc	P70	fav	*de novo*	Fc
P5	n.d.	n.d.	Fc	P38	adv	*de novo*	Fc	P71	int	*de novo*	Fc, mRNA
P6	adv	*de novo*	mRNA	P39	fav	*de novo*	Fc	P72	adv	sAML	Fc, mRNA
P7	adv	*de novo*	mRNA	P40	int	sAML	Fc, mRNA	P73	adv	*de novo*	mRNA
P8	int	R/R AML	Fc	P41	int	*de novo*	Fc	P74	int	R/R AML	Fc
P9	adv	sAML	Fc	P42	int	*de novo*	Fc, mRNA	P75	int	*de novo*	Fc
P10	int	*de novo*	Fc	P43	fav	R/R AML	Fc	P76	adv	*de novo*	mRNA
P11	adv	*de novo*	Fc	P44	int	R/R AML	Fc	P77	int	*de novo*	mRNA
P12	int	*de novo*	Fc, mRNA	P45	int	*de novo*	Fc, mRNA	P78	adv	*de novo*	mRNA
P13	fav	*de novo*	Fc	P46	int	*de novo*	Fc	P79	fav	sAML	mRNA
P14	int	sAML	Fc	P47	fav	*de novo*	mRNA	P80	int	sAML	mRNA
P15	fav	*de novo*	Fc	P48	adv	*de novo*	Fc	P81	int	R/R AML	mRNA
P16	int	*de novo*	Fc	P49	fav	*de novo*	Fc	P82	adv	*de novo*	mRNA
P17	fav	*de novo*	Fc	P50	int	sAML	Fc	P83	adv	*de novo*	mRNA
P18	n.d.	n.d.	Fc	P51	int	*de novo*	Fc	P84	adv	R/R AML	mRNA
P19	int	R/R AML	Fc	P52	int	*de novo*	Fc, mRNA	P85	fav	*de novo*	mRNA
P20	fav	*de novo*	Fc	P53	adv	sAML	Fc	P86	int	*de novo*	mRNA
P21	fav	sAML	Fc	P54	adv	sAML	Fc	P87	fav	R/R AML	mRNA
P22	fav	R/R AML	Fc	P55	adv	sAML	Fc	P88	fav	sAML	mRNA
P23	int	*de novo*	Fc	P56	adv	*de novo*	Fc	P89	int	*de novo*	mRNA
P24	int	*de novo*	Fc	P57	fav	*de novo*	mRNA	P90	int	*de novo*	mRNA
P25	fav	*de novo*	Fc	P58	n.d.	n.d.	Fc, mRNA	P91	adv	sAML	mRNA
P26	int	sAML	Fc, mRNA	P59	adv	*de novo*	Fc, mRNA	P92	adv	*de novo*	mRNA
P27	adv	*de novo*	mRNA	P60	adv	sAML	Fc, mRNA	P93	adv	*de novo*	mRNA
P28	adv	*de novo*	Fc	P61	fav	*de novo*	Fc, mRNA	P94	n.d.	n.d.	mRNA
P29	n.d.	n.d.	Fc	P62	adv	R/R AML	Fc	P95	fav	*de novo*	Fc
P30	n.d.	n.d.	Fc	P63	adv	sAML	Fc, mRNA	P96	adv	*de novo*	Fc
P31	adv	*de novo*	Fc	P64	int	*de novo*	Fc	P97	adv	*de novo*	Fc
P32	adv	*de novo*	mRNA	P65	adv	sAML	Fc	P98	fav	R/R AML	Fc
P33	adv	*de novo*	Fc	P66	adv	sAML	Fc	P99	n.d.	n.d.	Fc

Supplemental Table S1

Spec. = Specimen. adv = adverse risk, int = intermediate risk, fav = favorable risk profiles according to ELN 2017 criteria [6]; sAML = secondary AML (defined as AML progressing from MDS or MDS/MPN and therapy related AML according to the ICC 2022 classification [23]); R/R AML, relapsing or refractory AML according to ELN 2022 recommendations: Diagnosis and management of AML in adults [7]; n.d. = not determined; Fc = flow cytometry analysis; mRNA = *iASPP* mRNA expression levels analysis.
